# Vitamin B12-Loaded Chitosan Nanoparticles Promote Skeletal Muscle Injury Repair in Aged Rats via Amelioration of Aging-Suppressed Efferocytosis

**DOI:** 10.3390/biom15121709

**Published:** 2025-12-07

**Authors:** Walaa Bayoumie El Gazzar, Amina A. Farag, Heba Bayoumi, Shaimaa E. Radwaan, Lina Abdelhady Mohammed, Hend Elsayed Nasr, Nashwa E. Ahmed, Reham M. Ibrahim, Mahmoud Mostafa, Shimaa K. Mohamed, Dania Abdelhady, Eman E. Elwakeel, Amira M. Badr, Sahar Soliman

**Affiliations:** 1 Department of Medical Biochemistry and Molecular Biology, Faculty of Medicine, Benha University, Benha 13518, Egypt; lina.mohamed@fmed.bu.edu.eg (L.A.M.); hend.mosalm@fmed.bu.edu.eg (H.E.N.); nashwa.mohamed@fmed.bu.edu.eg (N.E.A.); 2 Department of Anatomy, Physiology and Biochemistry, Faculty of Medicine, The Hashemite University, P.O. Box 330127, Zarqa 13133, Jordan; 3 Department of Forensic Medicine and Clinical Toxicology, Faculty of Medicine, Benha University, Benha 13518, Egypt; amina.farag@fmed.bu.edu.eg; 4 Department of Histology and Cell Biology, Faculty of Medicine, Benha University, Benha 13518, Egypt; heba.bayoumi@fmed.bu.edu.eg; 5 Department of Zoology, Faculty of Science, Benha University, Benha 13518, Egypt; shaimaa.elsayed@fsc.bu.edu.eg; 6 Department of Physiology, Faculty of Medicine, Benha University, Benha 13518, Egypt; reham.mohamed@fmed.bu.edu.eg (R.M.I.); dania.mohamed@fmed.bu.edu.eg (D.A.); 7 Department of Pharmaceutics, Faculty of Pharmacy, Minia University, Minia 61519, Egypt; mahmoud_mohamed@mu.edu.eg; 8 Department of Pharmaceutics, Faculty of Pharmacy, Minia National University, New Minia 61768, Egypt; 9 Department of Pharmacology and Toxicology, Faculty of Pharmacy, Helwan University, Cairo 11795, Egypt; shimaa_kamal@pharm.helwan.edu.eg; 10 Department of Biomedical Science, Dubai Medical University, Dubai 19099, United Arab Emirates; 11 Department of Anatomy and Embryology, Faculty of Medicine, Benha University, Benha 13518, Egypt; eman.ismail@fmed.bu.edu.eg; 12 Department of Pharmacology and Toxicology, College of Pharmacy, King Saud University, P.O. Box 22452, Riyadh 11495, Saudi Arabia; amibadr@ksu.edu.sa; 13 Department of Physiology and Pharmacology, College of Osteopathic Medicine, Sam Houston State University, Conroe, TX 77304, USA

**Keywords:** vitamin B12, doxorubicin, efferocytosis, skeletal muscle, aging, muscle regeneration

## Abstract

Muscle gradually loses its regenerative capacity with aging. Recent evidence highlights age-related immune dysregulation as a key driver of satellite cell dysfunction and reduced muscle regeneration. Timely elimination of apoptotic cells by phagocytes through efferocytosis is essential for tissue repair. Therefore, exploring age-related alterations in the molecular machinery of efferocytosis and their impact on muscle regeneration is of great relevance. This study examined the efferocytic machinery in the gastrocnemius muscle tissue of young and aged rats after doxorubicin-induced acute myotoxicity and assessed the potential of Vitamin B12-loaded chitosan nanoparticles (B12 CS NPS) to enhance efferocytosis and promote skeletal muscle injury repair in aged rats. Aged rats exhibited impaired efferocytosis with a significant reduction in *MerTK*, *PPARγ*, and miR-124 expression, and increased *ADAM17* expression. B12 CS NPS administration significantly improved efferocytosis and reduced necrotic tissue areas, accompanied by increased *MerTK*, *PPARγ*, and miR-124, and reduced *ADAM17* expression. Supplementation with B12 CS NPS significantly enhanced satellite cell proliferation and differentiation, which was indicated by upregulated expression of *Pax7*, *Myog*, and *MyoD*. These findings reveal that age-related alterations in regulatory molecules impair efferocytosis in aged muscle and demonstrate the potential of B12 CS NPs to enhance efferocytosis and improve skeletal muscle repair.

## 1. Introduction

In modern societies, aging represents the greatest risk factor for most diseases. Yet, despite decades of research, the intricate nature of cellular and organismal aging continues to constrain our understanding of this fundamental biological process [1]. With aging, most tissues and organs gradually lose function, partly due to diminished stem cell-mediated regeneration. The skeletal muscle illustrates this trend. Its stem cells, known as satellite cells, maintain regenerative potential across the lifespan, but they exhibit impaired function in the elderly for primarily unresolved mechanisms [2]. Hence, the muscle undergoes a progressive, age-related decline in regenerative capacity after tissue injury, contributing to functional deficits associated with aging. While young muscles can typically restore muscle size and performance within a few weeks of suffering an acute injury, aged muscles often experience extended, and potentially lasting, deficits.

Age-driven impairments in muscle regenerative mechanisms may stem from internal muscle defects or from age-driven alterations in external regulatory networks [3]. In youthful organisms, satellite cell activity is finely tuned by niche and systemic signals. Surrounding milieu disruptions profoundly impact their quiescence, differentiation, and self-renewal, with extracellular milieu deterioration recognized as a key driver of age-related stem cell dysfunction. In particular, age-linked remodeling processes in the immune landscape have been increasingly acknowledged as major contributors to satellite cell deficits and hence the impaired regenerative potential of aged muscles. An effective regenerative outcome relies on a well-orchestrated immune-mediated response to muscle injury, engaging multiple immune cell populations and coordinating pro- and anti-inflammatory signaling [2].

Macrophages orchestrate the inflammatory response following tissue injury. The initial inflammatory process to a muscle injury is characterized by the early increase in M1 macrophages, which secrete inflammatory cytokines, such as tumor necrosis factor alpha (TNF-a) and Interleukin-1β (IL1β). These cytokines have powerful effects on myogenesis, supporting the role of M1 macrophages in satellite cell activation. The early infiltration of M1 macrophages is subsequently replaced by an enhanced presence of M2 macrophages, promoting tissue repair and satellite cell maturation. In vitro, M2-derived IL-4 and IL-10 support myoblast differentiation and drive myogenin (Myog) expression, required for satellite cell maturation. While acute inflammation supports effective skeletal muscle regeneration, chronic or unresolved inflammation—as seen in idiopathic inflammatory myopathies, muscular dystrophies, and aging—is linked to dysfunction of immune cell populations, satellite cells, and fibro–adipogenic progenitor cells (FAPs), driving fibrosis and poor skeletal muscle regeneration [3,4]. Thus, the timing and balance of M1 and M2 macrophages are key determinants of proper skeletal muscle repair. In addition, one of the primary functions of macrophages is to swiftly remove apoptotic cells through the orchestrated clearance mechanism called efferocytosis, which is essential for sustaining tissue homeostasis, resolving inflammation, and promoting repair [5,6]. In several chronic non-resolving inflammatory diseases, impaired efferocytosis gives rise to apoptotic cells accumulation. These cells may undergo secondary necrosis, promoting tissue damage and pathological inflammation. Defects in efferocytosis may drive the persistent low-grade inflammation associated with aging, commonly referred to as inflammaging [6]. Consequently, a key focus of biomedical studies is to elucidate the mechanisms underlying efficient efferocytosis in normal physiology and the factors leading to its dysfunction in disease [7]. The TAM family of receptor tyrosine kinases (MerTK, Axl, and Tyro3) is critical in regulating efferocytosis. Among them, Mer tyrosine kinase (MerTK) is extensively expressed across immune cell populations and is markedly upregulated in macrophages during the M1-to-M2 transition. In macrophages, it mediates the recognition of phosphatidylserine (PS) on the surface of dying cells through GAS6 (Growth-arrest-specific-6) and PROS1 (protein S1), promoting phagocytic clearance, inflammation resolution, and suppression of innate immune responses following acute injury. Deficiency in MerTK impairs apoptotic body clearance, leading to increased necrosis observed in conditions such as atherosclerosis and myocardial infarction [8]. Mertk is regulated post-translationally via shedding of the receptor’s extracellular domain, producing a signaling-incompetent receptor unable to respond to of Gas6- and PROS1-mediated activation. A Disintegrin and Metalloproteinase 17 (ADAM17), also known as TACE (TNF-α converting enzyme), is the best characterized enzyme mediating Mertk proteolytic cleavage [9].

At the transcriptional level, the peroxisome proliferator-activated receptor γ (PPARγ) is a key regulator that coordinates macrophage functions, with its signaling being essential for apoptotic cell clearance through upregulating efferocytosis-related molecules such as MerTK, and facilitating the transition from pro- to anti-inflammatory macrophages, thereby supporting inflammation resolution and tissue homeostasis [6,10]. PPAR-γ activation has additionally been shown to enhance miR-124 expression [11,12]. miR-124 is increasingly acknowledged as central to the modulation of immunity and inflammation, and has been shown to block ADAM17 expression by binding its 3′-UTR [13,14,15]. Nevertheless, alterations in MerTK due to aging and its role and underlying mechanisms in regulating efferocytosis and muscle regeneration remain unclear. Therefore, we sought to unravel potential molecular mechanisms that could be harnessed to enhance efferocytosis and muscle regeneration during aging.

Recently, vitamin B12 (Vit B12), by virtue of its fundamental functions in one-carbon metabolism and epigenetic mechanisms, has been demonstrated as a key player during cellular reprogramming and tissue repair [16]. In addition, Vit B12 is proposed to have a crucial role in modulating the immune system, but the detailed molecular mechanisms remain poorly understood [17,18]. Hence, we found it intriguing to study the impact of acute, post-injury supplementation of Vit B12 on the immune compartment, through investigating its effect on efferocytosis and macrophage phenotypic shift, and how this may influence muscle regeneration during aging.

Acute skeletal muscle injury was modeled using doxorubicin, an anthracycline-type anticancer agent known as a potent inducer of apoptosis in muscle cells, particularly in aged tissues [19,20,21,22].

Given the unique role of the immune system in tissue repair and regeneration, and that prompt clearance of apoptotic cells through efferocytosis is crucial for avoiding excessive inflammation and tissue injury, we hypothesized that age-related alterations in the molecular machinery of efferocytosis may contribute to reduction in the regenerative capacity of muscles during aging. Therefore, we assessed whether aging affects the efferocytic process through alterations in the efferocytic receptor MerTK and its potential underlying regulatory molecules such as ADAM17, PPARγ, and miR-124. We also assessed the age-related changes in macrophage polarization status at an early time point, day 3, after acute injury. Age-related changes in the population of satellite cells at this time point after injury were evaluated by histological analysis and the expression of Pax7, MYOD, and Myog.

Importantly, because no previous work has investigated how Vit B12 influences efferocytosis in aged skeletal muscles, we further explored whether Vit B12 administration could be a feasible therapeutic approach to enhance muscle tissue regeneration during aging by promoting efferocytosis and regulating the balance of M1/M2 macrophage polarization.

## 2. Materials and Methods

### 2.1. Preparation and Characterization of Vit B12-Loaded Chitosan Nanoparticles (B12 CS NPS)

Chitosan nanoparticles (CS NPs) were synthesized using a modified ionic gelation method [23]. A 2 mg/mL chitosan (CS) solution was prepared by dissolving chitosan in 0.1% (*v*/*v*) acetic acid solution diluted with double-distilled water. Separately, a 2.5 mg/mL solution of sodium tripolyphosphate (TPP) was prepared by dissolving an appropriate quantity of TPP in double-distilled water under constant stirring. Once fully dissolved, Vit B12 was added to the TPP solution, and the mixture was homogenized. The resulting solution containing TPP and VB12 was gradually added dropwise into the CS solution under continuous stirring for 3 h, maintaining a final CS/TPP ratio of 3:1.

The entrapment efficiency of Vit B12 was determined by centrifuging 1 mL of the prepared nanoparticle formulation at 12,000 rpm for 60 min, followed by removal of the supernatant. The pellet was re-dispersed in double-distilled water and subjected to two additional rounds of centrifugation under the same conditions. The resulting pellet was dissolved in methanol, sonicated, and vortexed for 20 min. After centrifugation at 12,000 rpm for 5 min, the supernatant was collected, diluted, and analyzed spectrophotometrically at 362 nm to quantify the Vit B12 content. The prepared B12 CS NPS were characterized for particle size, zeta potential, surface morphology, and drug in vitro release as previously reported [24].

### 2.2. Animals

Young (2 month) and aged (22 month) male Wistar albino rats were purchased from Helwan farm for experimental animals, VACSERA, Cairo, Egypt. Rats were divided into six groups of seven, each housed in separate cages under controlled conditions (23 °C  ±  2 °C, 45 ± 5% humidity, and 12/12 h light and dark cycles) with free access to food and water. Animals were fed a basal diet (AIN-93M) [25], purchased from El-Gomhouria Pharmaceutical Company, Cairo, Egypt. All animals were acclimated for one week before the experiment. The study was conducted in accordance with established guidelines for the care and use of laboratory animals [26], and was approved by the Animal Care and Use Committee, Zoology Department, Faculty of Science, Benha University, Egypt (Approval No: ZD/FSc/BU-IACUC/2025-28).

### 2.3. Chemicals

Doxorubicin HCl (DOXO) (Adricin^®^) was purchased from Hikma pharmaceuticals (Cairo, Egypt). Vitamin B12 (Cyanocobalamin), chitosan (CS) (molecular weight 100,000–300,000) and Tripolyphosphate (TPP), were purchased from Sigma-Aldrich (Sigma, St. Louis, MO, USA). Acetic acid solution was purchased from El-Nasr Pharmaceutical Chemicals Company (Cairo, Egypt).

### 2.4. Study Design

The animals were divided into the following groups:

Group I (Young control group; *n* = 7): On day 0, this group received a single intraperitoneal injection of normal saline.

Group II (Old control group; *n* = 7): On day 0, this group received a single intraperitoneal injection of normal saline.

Group III (Young + DOXO; *n* = 7): On day 0, this group received a single intraperitoneal injection of DOXO (20 mg/kg) [19,27,28].

Group IV (Old + DOXO; *n* = 7): On day 0, this group received a single intraperitoneal injection of DOXO (20 mg/kg).

Group V (Old + B12 CS NPS; *n* = 7): Beginning 6 days before day 0, this group was given intramuscular injections of Vit B12-loaded chitosan nanoparticles (13.5 mg/kg) [29], with one administered every three days. On day 0, the animals also received a single intraperitoneal injection of normal saline.

Group VI (Old + DOXO + B12 CS NPS; *n* = 7): Starting 6 days before day 0, this group was given intramuscular injections of Vit B12-loaded chitosan nanoparticles (13.5 mg/kg), with one administered every three days. On day 0, they also received a single intraperitoneal injection of DOXO (20 mg/kg).

**Figure 1:**
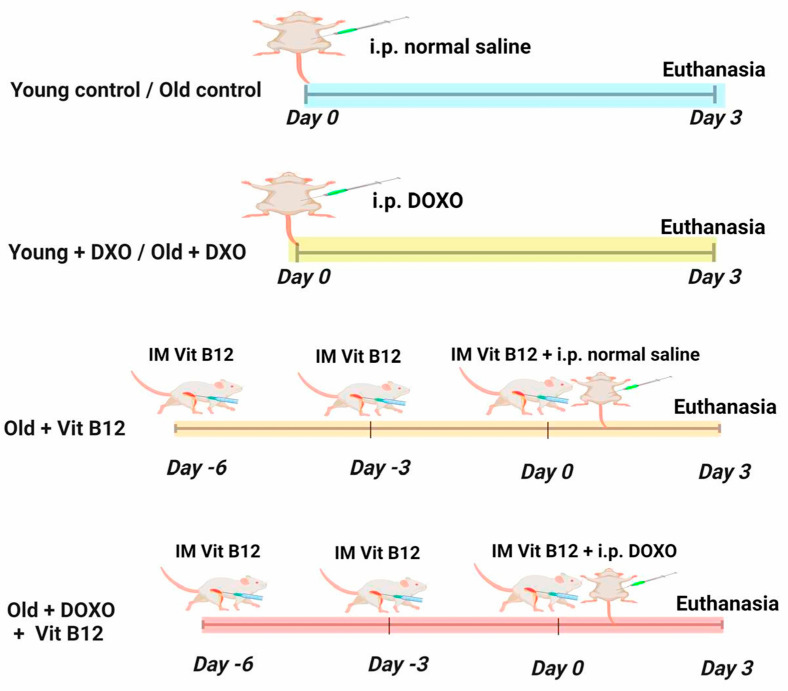
Schematic representation summarizing the study design. Six groups of rats (*n* = 7 per group) were used, and gastrocnemius muscles were collected for biochemical, histological, and molecular analyses.

At the end of day 3, after the DOXO challenge, all rats underwent euthanasia via decapitation subsequent to isoflurane inhalation anesthesia (El Amriya for pharmaceutical industries, Al Amyria, Alexandria). Figure 1 is a schematic representation summarizing the study design.

The gastrocnemius muscle of each rat was harvested bilaterally. The left gastrocnemius muscle was used for biochemical and PCR analysis. The right gastrocnemius muscle was used for histopathological and immunohistochemical examination. Small pieces, about (1 mm^3^), were put in 2.5% solution of glutaraldehyde on 0.1 M phosphate buffer (pH  =  7.4) mixed with 4.0% paraformaldehyde as a primary fixative for electron microscopy tissue processing. The remaining gastrocnemius muscle was immersed in 10% neutral buffered formalin for paraffin block preparation.

### 2.5. Biochemical Analysis

The gastrocnemius muscle tissue samples were flushed with cold saline, homogenized in phosphate buffer (pH 6–7) using a mixer mill MM400 (Retsch, Haan, Germany), and centrifuged at 4000 rpm for 15 min at 4 °C. The resulting supernatant was then used to quantitatively assess the following pro-inflammatory and anti-inflammatory cytokines, and oxidative stress markers per the manufacturer: Interleukin-1β (IL1β) utilizing Rat IL-1β ELISA Kit (Catalog #: E-EL-R0012; Elabscience, TX, USA), tumor necrosis factor (TNF-α) utilizing rat tumor necrosis α ELISA Kit (Catalog #: RAB0480; Sigma-Aldrich, St. Louis, MO, USA), interleukin 6 (IL-6) utilizing rat IL-6 ELISA Kit (Catalog #: E-EL-R0015; Elabscience, TX, USA), interleukin 10 (IL-10) utilizing Rat IL-10 ELISA Kit (Catalog #: E-EL-R0016; Elabscience, TX, USA), total antioxidant capacity (TAC) (Catalog # TA 25 13; BioDiagnostic, Giza, Egypt) and malondialdehyde (MDA) (Catalog # MD 25 29; BioDiagnostic, Giza, Egypt).

### 2.6. Quantitative Real-Time Polymerase Chain Reaction (qPCR) Analysis for mRNA Gene Expression of BAX, Bcl-2, MerTK, ADAM17, PPARγ, MiR-124, Pax7, MyoD and Myog

Samples of the gastrocnemius muscle tissues utilized for RNA extraction and qPCR were preserved in RNAlater™ stabilization solution (Catalog No: AM7021; Thermo Fisher Scientific, MA, USA) at 10 µL per 1 mg of tissue; then, samples were frozen at −80 °C for subsequent RNA extraction and purification.

#### 2.6.1. Total RNA Extraction

Total RNA extraction from the gastrocnemius muscle tissue samples was performed using RNeasy Mini Kit (Catalog No: 74104; Qiagen, Hilden, Germany). RNA purity and concentration were evaluated utilizing a NanoDrop^®^ ND–1000 spectrophotometer (NanoDrop Technologies; Wilmington, DE, USA).

#### 2.6.2. SYBR Green RT-qPCR

The real-time RT-PCR was performed in the StepOneTM real-time PCR machine (Life technologies, MA, USA) using HERA SYBR^®^ Green RT-qPCR Kit (Catalog No: WF10303001; Willowfort, UK) following the manufacturer’s instructions. The oligonucleotide specific primers were supplied from Metabion (Planegg, Germany), displayed in Table 1.

The PCR cycling conditions included reverse transcription at 50 °C for 30 min and primary denaturation at 94 °C for 15 min followed by 40 cycles; each process entails denaturation at 94 °C for 15 s, annealing at 60 °C for 30 s, and extension at 72 °C for 30 s.

For mRNA targets, expression levels were normalized to β-actin. For miRNA analysis, miR-124 expression was quantified separately and normalized to U6 snRNA. Relative expression was calculated using the 2−ΔΔCt method [30] and expressed as the n-fold changes relative to controls.

### 2.7. Histological Analysis

Following 72 h fixation in 10% neutral buffered formalin, gastrocnemius tissues were trimmed, dehydrated in serial ethanol concentrations, xylene-cleared, wax-infiltrated, and embedded in Paraplast. Sections of 5 µm were obtained via rotary microtome, mounted on slides, and stained with H&E (routine histology) and Masson’s trichrome (for collagen fibers), then examined blindly by a skilled histologist. All standard procedures for samples fixation, processing, and staining were performed according to Bancroft and Layton (2019) [31]. Slides were examined blindly on ×200 and ×400 magnification.

### 2.8. Immunohistochemical Analysis

According to standard procedures [32], deparaffinized 5-micron thick tissue sections were cut and prepared, and the tissue sections were treated by 3% hydrogen peroxide for 20 min, washed by PBS, then incubated with anti-Caspase-3 as a marker for apoptosis (active/pro) (1: 200- Clone 31A1067, Catalog # MC0123, Medaysis, Livermore, CA, USA), anti-iNOS monoclonal antibody (Clone SP126; Springbio, CA, USA), anti-CD86 primary antibody as a marker for M1 microglial phenotype (bs-1035R, BIOSS, MA, USA—1:150), anti-CD163 antibody as a marker for M2 microglial phenotype (GeneTex. GTX35247 1:100), left overnight at 4C, washed with PBS and incubated with the secondary antibody using the HRP Envision kit (DAKO, Glostrup, Denmark) for 20 min. After another PBS wash, the slides were treated with diaminobenzidine (DAB) for 10 min, followed by PBS washing. Counterstaining was performed with hematoxylin, after which the sections were dehydrated, cleared in xylene, and cover-slipped for microscopic examination.

### 2.9. Microscopic Analysis

One slide was prepared for each rat. Six random non overlapping skeletal muscle fields were analyzed per tissue section of each sample. Fields were examined at ×400 magnification for H&E, CD86 and CD163, and at ×200 for H&E, iNOS, caspase-3, and Masson’s trichrome to assess inflammation, relative area% of immunohistochemical expression of caspase-3, iNOS, and Masson’s trichrome, as well as mean reactive positive macrophages for CD86 and CD163.

### 2.10. Computer-Assisted Digital Image Analysis (Digital Morphometric Study)

Slides were photographed using an Olympus^®^ digital camera installed on an Olympus^®^ microscope (Tokyo, Japan) with 0.5× photo adaptor, using ×20 and ×40 objective lenses and saved as TIFF. The resulting images were analyzed using an Intel^®^ Core I7^®^-based computer using VideoTest Morphology^®^ software 4.0 (Russia) with a specific built-in routine for particle analysis. Results were exported to Excel Sheet expressed as% positively reactive cells for CD86 and CD163, and the area% for caspase-3, iNOS immune expression, as well as Masson’s trichrome stain.

### 2.11. Transmission Electron Microscopic Examination

After primary fixation in a 2.5% glutaraldehyde solution prepared in 0.1 M phosphate buffer (pH 7.4) mixed with 4.0% paraformaldehyde, the fixative was removed by rinsing the samples three times with distilled water for 10 min each. Then, the samples were post-fixed in 1.0% osmium tetraoxide solution for 2 h. After fixation, the tissue was washed, dehydrated, and embedded in fresh resin overnight at 60 °C. Semithin Sections (0.5–1 μm) were cut, stained with toluidine blue, and examined at magnification ×1000. Ultrathin slices (70–90 nm) were cut using ultra-microtome and stained with double staining technique of uranyl acetate followed by lead citrate. Tissue evaluation of the sections was performed at Faculty of Agriculture, (Cairo University Research Park’s (CURP) electronic microscope lab using transmission electron microscopy (JEM—1400, JEOL model—Tokyo, Japan) operated at 80 kV. The protocol was performed according to (Glauert and Lewis, 1998) [33].

### 2.12. Statistical Analysis

To compare the means among various groups for the measured parameters, we first tested the distribution of each variable using Q-Q plots and Shapiro test and then parametric one-way ANOVA was used followed by Tukey’s multiple comparison test. The differences among groups were considered statistically significant if the adjusted *p*-value was <0.05, with the degree of the significance being assessed using the following thresholds *: <0.05, **: <0.01, ***: <0.001, ****: <0.0001. Statistical analysis and data visualization were performed using GraphPad Prism version 9 (San Diego, CA, USA).

## 3. Results

### 3.1. Preparation and Characterization of B12 CS NPS

**Figure 2:**
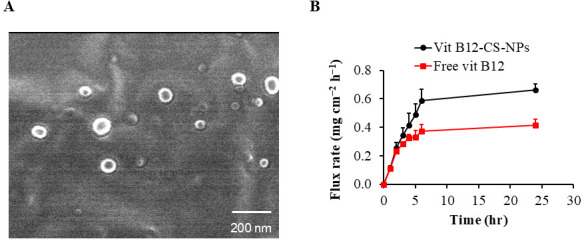
Characterization of B12 CS NPS. (**A**) Scanning electron microscopy examination showing the spherical nature of the B12-CS-NPS. (**B**) In vitro release analysis of Vit B12 from chitosan nanoparticles.

B12-CS-NPS were successfully developed using the ionic gelation method. Particle size, polydispersity index, zeta potential, encapsulation efficiency, scanning electron microscopy, and in vitro release tests were used to analyze the produced systems. The average particle diameter of the produced particles was measured with Zetasizer Nano and found to be 93.13 ± 3.6 nm. A polydispersity index of 0.261 for the nanoparticles suspension also suggested uniform particle dispersion. Moreover, nanoparticulate systems show a somewhat negative surface charge of +43.8 ± 7.54 mV for their zeta potential. Scanning electron microscopy photographs showed that the B12-CS-NPS were approximately spherical, with an average particle diameter that was similar to what was found using Zetasizer nano analysis (Figure 2A). The nanoparticulate systems’ entrapment efficiencies were determined to be 22.65 ± 1.61%. Figure 2B shows the cumulative release rate of Vit B12 from chitosan nanoparticles based on the in vitro release analysis. Free Vit B12 increased with flux rate, equal to 0.37 mg cm^−2^ h^−1^ in 6 h and 0.416 mg cm^−2^ h^−1^ in 24 h during the release study. Vit B12 release continued as it was encapsulated in the chitosan nanoparticles, reaching 0.589 mg cm^−2^ h^−1^ and 0.662 mg cm^−2^ h^−1^ after 6 and 24 h of release study, respectively.

### 3.2. Aging Suppressed Efferocytosis of Apoptotic Skeletal Muscle Cells

We initially assessed the efficiency of apoptotic cell efferocytosis in young versus aged groups. This was accomplished by assessing apoptosis in skeletal muscle cells through evaluating the apoptotic markers Bax, Bcl-2, and cleaved caspase-3, assessing necrotic tissue area, spacing between muscle fibers, fiber average area, and demonstrating efferocytosis signs of by EM.

**Figure 3:**
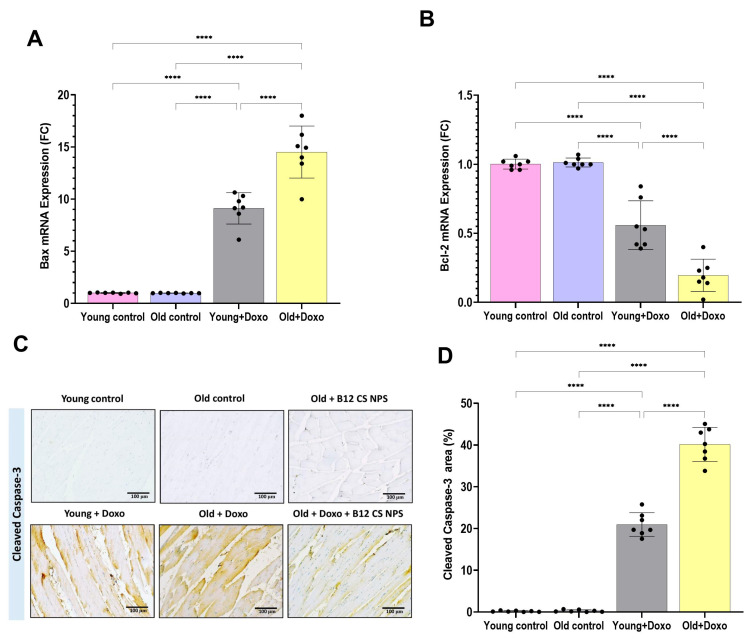
(**A**) BAX and (**B**) BCL-2 gene expression in the gastrocnemius muscle tissue samples from experimental groups (*n* = 7/group), as determined by qPCR, normalized for the house-keeping gene B-actin, and expressed relative to controls. Results are expressed as mean ± SD. ****, significant at (*p* < 0.0001). (**C**) Representative photomicrographs showing immunohistochemical detection of caspase-3 (counterstained with hematoxylin). (**D**) Quantitative representation of cleaved caspase-3 area%.

Aged rats treated with Doxo exhibited a significant increase in mRNA expression of the pro-apoptotic Bax gene (Figure 3A) (mean difference [young + Doxo–Old + Doxo] = −5.4, 95% CI: −7.546 to −3.254, *p* < 0.0001), along with heightened caspase-3 immunoreactivity (Figure 3C,D) (mean difference [young + Doxo – Old + Doxo] = −19.21, 95% CI: −22.88 to −15.54, *p* < 0.0001), coupled with a marked reduction in mRNA expression of the anti-apoptotic Bcl-2 gene (Figure 3B) (mean difference [young + Doxo – Old + Doxo] = 0.3629, 95% CI: 0.2025 to 0.5232, *p* < 0.0001) compared with young rats treated with Doxo.

**Figure 4:**
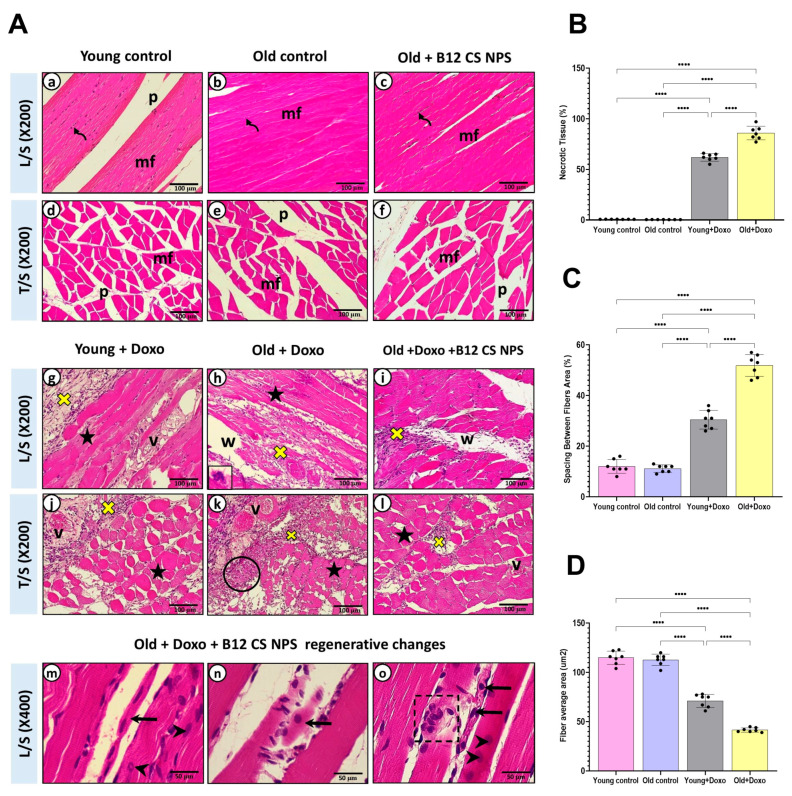
(**A**) Photoplate (**a**–**l**) representing L/S and T/S of skeletal muscles from different study groups stained with (H&E) at magnification ×200. (**m**–**o**) Representing L/S sections from Old + Doxo + B12-CS NPS group with higher magnification (×400). (**B**) Necrotic tissue area, (**C**) spacing between muscle fibers, and (**D**) fiber average area in the gastrocnemius muscle tissue samples from experimental groups (*n* = 7/group). Results are expressed as mean ± SD. ****, significant at (*p* < 0.0001).

Histopathological examination showed that both young (Figure 4A(a,d)) and old control groups (Figure 4A(b,e)), exhibited normal architecture with intact myofibers (mf), peripheral flat nuclei (curved↑), and well-organized perimysium connective tissue (p). Aged rats treated with B12 CS NPS also displayed preserved histological features similar to controls (Figure 4A(c,f)). In contrast, young rats treated with Doxo exhibited massive inflammatory cell infiltration (x), congested vasculature (v), and widespread necrotic fibers (★) (Figure 4A(g,j)). These changes were more severe in aged rats treated with Doxo, which showed extensive neutrophil infiltration (lower left inset), completely degenerated fibers (circle), and markedly widened inter-fiber spaces (w) (Figure 4A(h,k)). Quantitative morphometry confirmed these findings, showing significantly increased necrotic area (Figure 4B), greater fiber spacing (Figure 4C), and reduced fiber cross-sectional area (Figure 4D).

**Figure 5:**
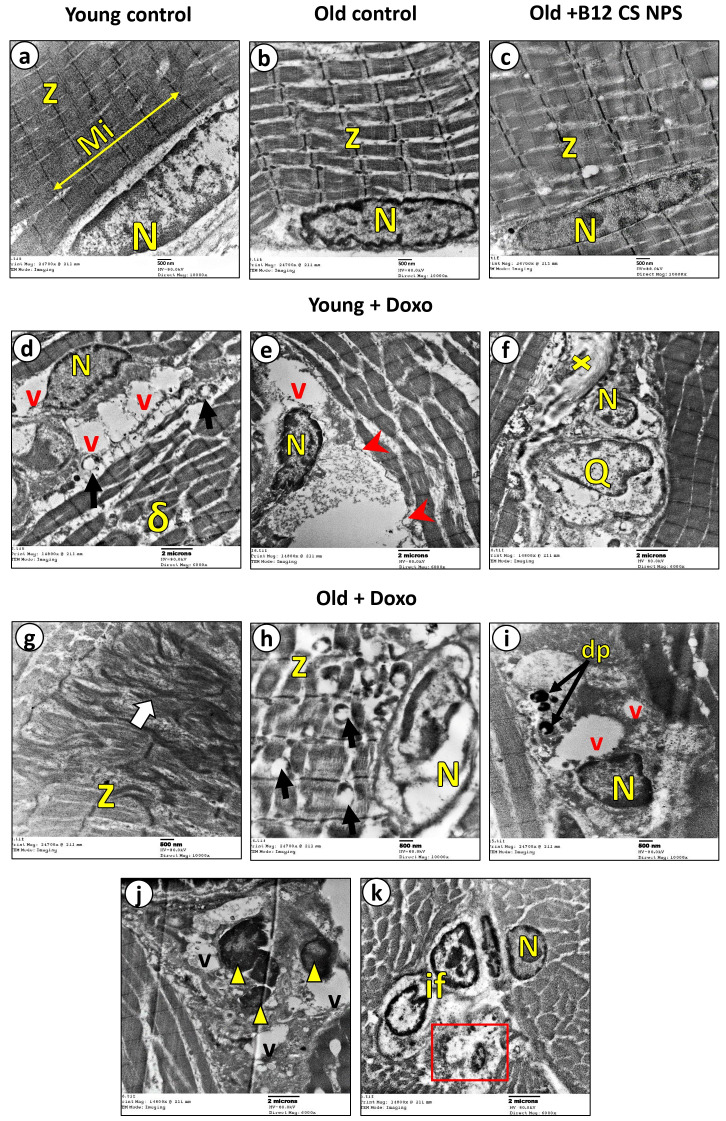
Representativetransmission electron photomicrographs (**a**–**k**) of skeletal muscle from the five study groups: Young control (**a**), old control (**b**), Old + B12-CS NPS (**c**), Young + Doxo group (**d**–**f**), and Old + Doxo group (**g**–**k**).

Ultrastructural evaluation showed that the young control group (Figure 5a), old control group (Figure 5b), and Old + B12-CS NPs (Figure 5c) groups all exhibited normal ultrastructure with sarcoplasm filled with parallel myofibrils (Mi), regularly arranged sarcomeres bounded by dark regular Z-lines, and peripherally located oval nuclei (N). Similarly, the old control group (b) and Old + B12-CS NPS group (c) displayed the same normal histological features as the control group, without pathological changes. Young rats treated with Doxo showed shrunken nuclei (N) surrounded by vacuolated sarcoplasm (v), vacuolated mitochondria (↑), and the myofibrils appeared disoriented and hypercontracted (δ) (Figure 5d). They also showed bizarrely shaped nucleus (N) within debris-filled regions, surrounded by vacuoles (v), with nearby basal lamina showing scalloping (arrowheads). The myofibrils were widely separated (Figure 5e). Areas of necrotic tissue (x) were invaded by large macrophages (Q) with irregular outlines in close contact with shrunken nuclei (N) (Figure 5f).

**Figure 6:**
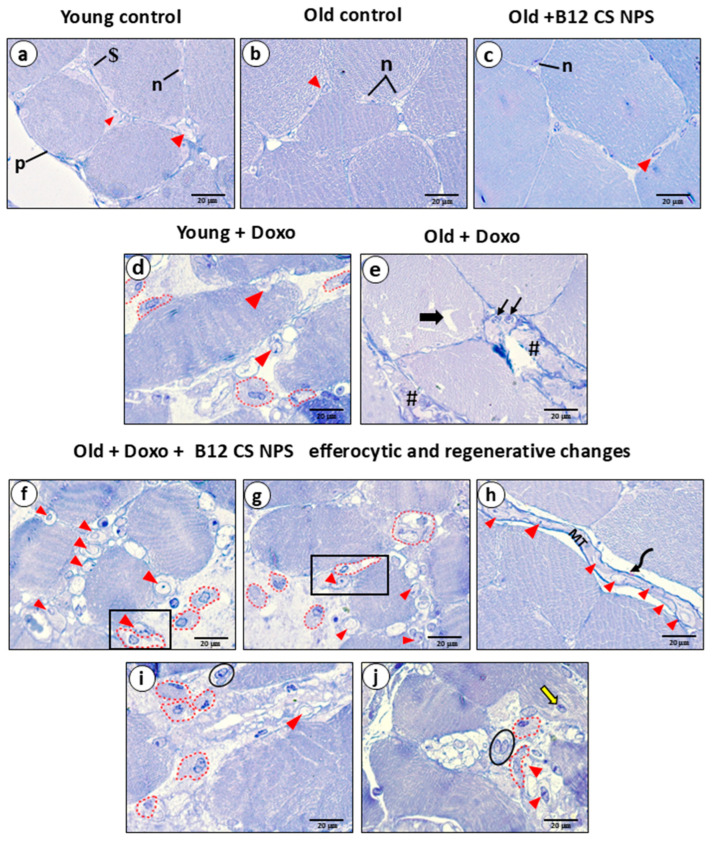
A photoplate (**a**–**j**) representing sections of skeletal muscles from different study groups stained with (toluidine blue) at magnification ×1000. Young control group (**a**): C/S showing myofibers with peripheral flat nuclei (n) beneath the basal lamina ($), while the satellite cells are located between the fibers outside the basal lamina (▲). The perimysium surrounds the bundle fibers (p). Old control (**b**) and Old + B12-CS NPS (**c**) displayed the normal histological features as control group. After induction of doxorubicin toxicity, Young + Doxo group (**d**) showed large spacing between fibers, these spaces are invaded by inflammatory cells, especially macrophages, which appeared large with irregular nuclei (dotted lines). Some satellite cells were apparent (▲). The Old + Doxo group (**e**) showed displaced myofibrils (thick ↑). Some fibers appeared as necrotic tissue (#), with apoptotic nuclei (↑). Old + Doxo + B12-CS NPS group (**f**–**j**) showed many efferocytic and regenerative features. (**f**,**g**) The areas between fibers are occupied by large numbers of satellite cells with euchromatic pale nuclei (▲) invading macrophages displaying irregular outlines (dotted lines). In many sites, the macrophages are located coupled with satellite cells (rectangles). (**h**) Prominent feature of newly formed myotube (MT) with aligned nuclei (▲) sealed with new basal lamina (curved arrow) was noticed. (**i**,**j**) Some active proliferating satellite cells can be noticed with two adjacent nuclei (oval) and some fibers showed centrally located nuclei (yellow ↑).

In contrast, aged rats treated with Doxo (Figure 5g–k) showed more inflammatory and apoptotic features at many sites. The sarcoplasm displayed highly distorted myofibrils alignment (white ↑) with irregular z-lines (Figure 5g). The sarcoplasm displayed abnormal dissolute nucleus (N), degenerated mitochondria (↑), and distorted z-lines (z) (Figure 5h). Some necrotic areas showed small shrunken nuclei with marginated heterochromatin (N), many vacuoles (v), and electron-dense apoptotic bodies (dp) (Figure 5i). Apoptotic nuclei appeared fragmented into small parts (▲) surrounded by multiple vacuoles (v) (Figure 5j). A necrotic area with very small apoptotic nucleus (rectangle), surrounded by inflammatory cellular infiltrate (if) was present, while the adjacent nucleus appeared shrunken (N) (Figure 5k). No obvious phagocytic activity of the necrotic tissue by macrophages was detected. Toluidine blue staining further confirmed the above features (Figure 6). These results collectively indicate that aging impairs macrophage-mediated efferocytosis, resulting in the accumulation of apoptotic and necrotic skeletal muscle cells post-doxorubicin-induced acute myotoxicity.

### 3.3. Suppressed MerTK and Enhanced ADAM17 Expression Contributed to Age-Related Impaired Efferocytosis

**Figure 7:**
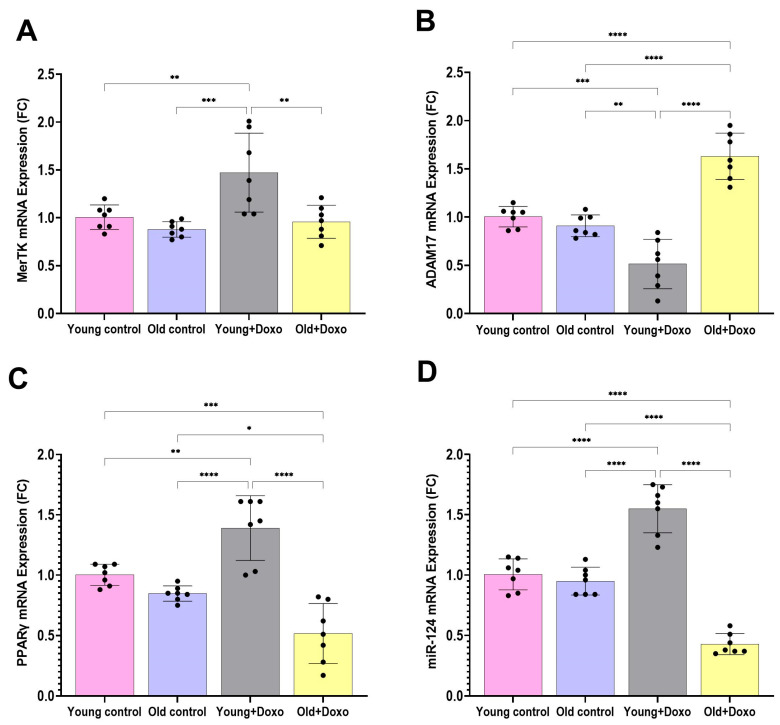
(**A**) MerTK, (**B**) ADAM17, (**C**) PPARγ, and (**D**) miR-124 gene expression in the gastrocnemius muscle tissue samples from experimental groups (*n* = 7/group), as determined by qPCR, normalized for the house-keeping gene B-actin, and expressed relative to controls. Results are expressed as mean ± SD. *, significant at (*p* < 0.05); **, significant at (*p* < 0.01), ***, significant at (*p* < 0.001) and ****, significant at (*p* < 0.0001).

To evaluate whether dysregulation of MerTK and its regulatory protease ADAM17 contributes to impaired efferocytosis in aging, we assessed their mRNA expression in the gastrocnemius tissue. No statistically significant age-related differences in MerTK mRNA expression were detected between aged and young control rats, although a trend toward a decrease was observed in the aged rats control group, while aged rats treated with Doxo exhibited a significant decrease in MerTK compared with young rats treated with Doxo (Figure 7A) (mean difference [young + Doxo–Old + Doxo] = 0.5129, 95% CI: 0.1648 to 0.8609, *p* < 0.01).

Moreover, a statistically significant decrease in ADAM17 mRNA expression was demonstrated in young rats treated with Doxo compared with controls (mean difference [young control–young + Doxo] = 0.4914, 95% CI: 0.2085 to 0.7743, *p* < 0.001), (mean difference [old control–young + Doxo] = 0.3971, 95% CI: 0.1143 to 0.6800, *p* < 0.01), while a significant increase in ADAM17 mRNA expression was observed in aged rats treated with Doxo compared with young rats treated with Doxo (mean difference [young + Doxo–Old + Doxo] = −1.117, 95% CI: 0.2085 to 0.7743, *p* < 0.0001) and with controls (mean difference [young control–old + Doxo] = −0.6257, 95% CI: −0.9086 to −0.3428, *p* < 0.0001), (mean difference [old control–old + Doxo] = −0.72, 95% CI: −1.003 to −0.4371, *p* < 0.0001) (Figure 7B).

### 3.4. PPARγ and MiR-124 Contribute to Age-Related Impaired Efferocytosis

To learn more about the potential molecular mechanisms governing mertk expression and explore their potential age-related alterations, we assessed PPARγ and miR-124 expression. Both have been shown to affect mertk and ADAM17 expression, and enhance clearance of apoptotic cells by macrophages. Interestingly, aged rats treated with Doxo exhibited a significant decrease in PPARγ (mean difference [young + Doxo–Old + Doxo] = 0.8729, 95% CI: 0.5922 to 1.154, *p* < 0.0001) (Figure 7C) and miR-124 expression (mean difference [young + Doxo–Old + Doxo] = 1.121, 95% CI: 0.9169 to 1.326, *p* < 0.0001) (Figure 7D) compared with young rats treated with Doxo.

### 3.5. Vit B12 Enhances Aging-Impaired Efferocytosis of Apoptotic Skeletal Muscle Cells

Given prior evidence supporting the regenerative benefits of Vit B12. We reasoned that Vit B12 administration throughout the repair and regeneration period may promote aging-impaired efferocytosis, thus improving tissue regeneration in aged rats after muscle injury. To address this idea, we assessed the effect of Vit B12 administration on the expression of the apoptotic markers (Bax, BCL-2, and cleaved caspase-3), along with necrotic tissue area, spacing between muscle fibers and fiber average area, and further demonstrated efferocytosis signs by EM in the aged rat groups.

**Figure 8:**
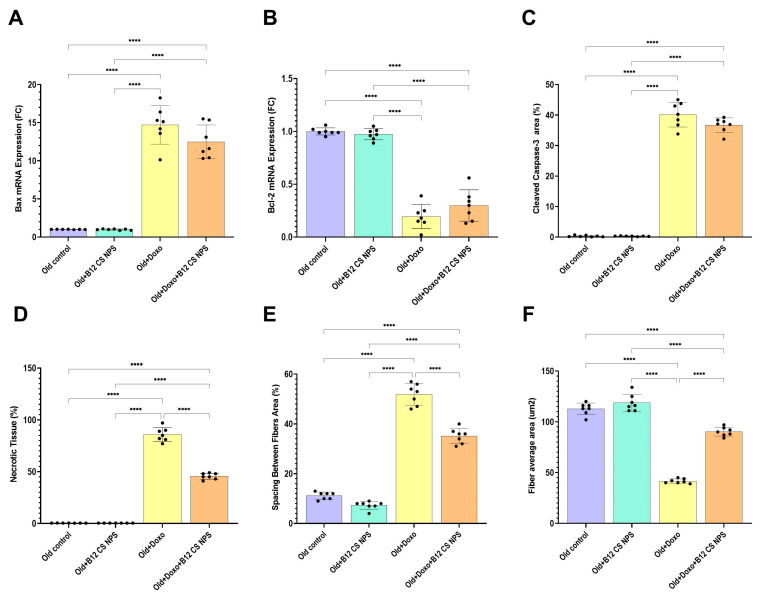
(**A**) BAX and (**B**) BCL-2 gene expression in the gastrocnemius muscle tissue samples from experimental groups (*n* = 7/group), as determined by qPCR, normalized for the house-keeping gene B-actin, and expressed relative to controls. (**C**) Quantitative representation of cleaved caspase-3 area%. (**D**) Necrotic tissue area, (**E**) spacing between muscle fibers, and (**F**) fiber average area in the gastrocnemius muscle tissue samples from experimental groups (*n* = 7/group). Results are expressed as mean ± SD. ****, significant at (*p* < 0.0001).

**Figure 9:**
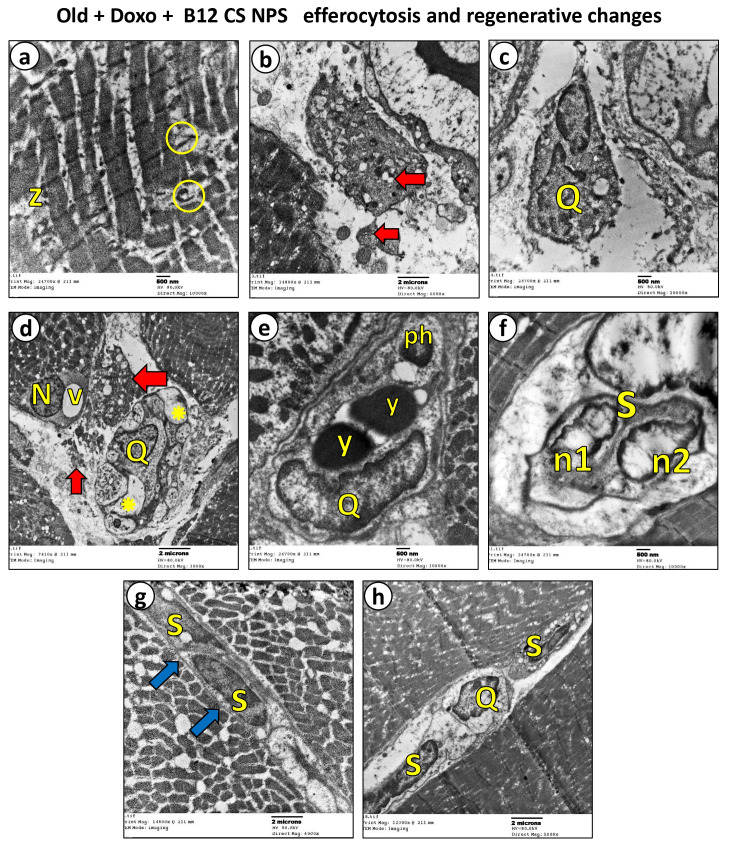
A photoplate (**a**–**h**) representing electron micrographs of skeletal muscles from Old + Doxo + B12-CS NPS group. When comparing Old + Doxo + B12-CS NPS group (**a**–**h**) to the previously described Old + Doxo group (Figure 5g–k), we found less inflammatory features accompanied by efferocytic and regenerative changes and (**a**) distorted and widely spaced myofibrils with vacoulated mitochondria (circles), and less-aligned z-lines (z). (**b**) Large apoptotic bodies are detached from the surrounding necrotic muscle tissue (red ↑). Many features of phagocytic activity indicate efferocytosis: (**c**) large macrophage with irregular nucleus (Q) appear to be invading the necrotic area. (**d**) Another large irregular macrophage displaying irregular nucleus (Q) at the site of inflammation near the detached necrotic debris (red ↑), showing multivesicular bodies as remnants of phagocytic activity (✸). The adjacent sarcoplasm showing shrunken nucleus (N) and large vacuole (v). (**e**) Large macrophages (Q) displayed well developed lysosomal apparatus (y) and phagosomes (ph) representing engulfed apoptotic bodies. Additionally, some features of regeneration are obvious. (**f**) Large satellite cell (S) in active stage of dividing nucleus into two small nuclei (n1 and n2). (**g**) Two adjacent satellite nuclei (S) are forming myotube (blue ↑) beneath the basal lamina representing proliferative activity. (**h**) Large macrophage (Q) with irregular nucleus in close contact with adjacent satellite cells (S).

Although Vit B12 decreased Bax gene expression (Figure 8A), enhanced Bcl-2 expression (Figure 8B) and attenuated caspase-3 immunoreactivity (Figure 3C and Figure 8C) in the aged rat group treated with Doxo + B12 CS NPS compared with the aged rat group treated with Doxo, the differences were not statistically significant. However, the intensity of inflammatory cells, the necrotic changes, and the congested vasculature were reduced compared to aged rats treated with Doxo (Figure 4A(i,l)). Additionally, a significant decrease in the necrotic tissue area (mean difference [old + Doxo–old + Doxo + B12 CS NPS] = 40.29, 95% CI: 34.90 to 45.67, *p* < 0.0001) (Figure 8D), and spacing between muscle fibers (mean difference [old + Doxo–old + Doxo + B12 CS NPS] = 16.71, 95% CI: 12.50 to 20.93, *p* < 0.0001) (Figure 8E) with increased fiber average area (mean difference [old + Doxo–old + Doxo + B12 CS NPS] = −48.71, 95% CI: −57.03 to −40.39, *p* < 0.0001) (Figure 8F) was demonstrated. Furthermore, the ultrastructure examination of the aged rat group treated with Doxo + B12 CS NPS compared with the aged rat group treated with Doxo revealed high efferocytic activity. Many macrophages with dense lysosomes were recorded invading the inflamed tissue and appeared in contact with tissue debris and fragmented apoptotic nuclei. Some macrophages also showed multivesicular bodies as remnants of phagocytic activity (Figure 9). These results suggest that efferocytosis functions more efficiently with Vit B12 administration, as even when in the presence of apoptosis, apoptotic cells are eliminated before progressing to necrosis, thereby clearly demonstrating the beneficial effect of Vit B12 enhancing efferocytosis.

### 3.6. Vit B12 Promotes Aging-Impaired Efferocytosis via Enhancing MerTK Expression and Supressing ADAM17

**Figure 10:**
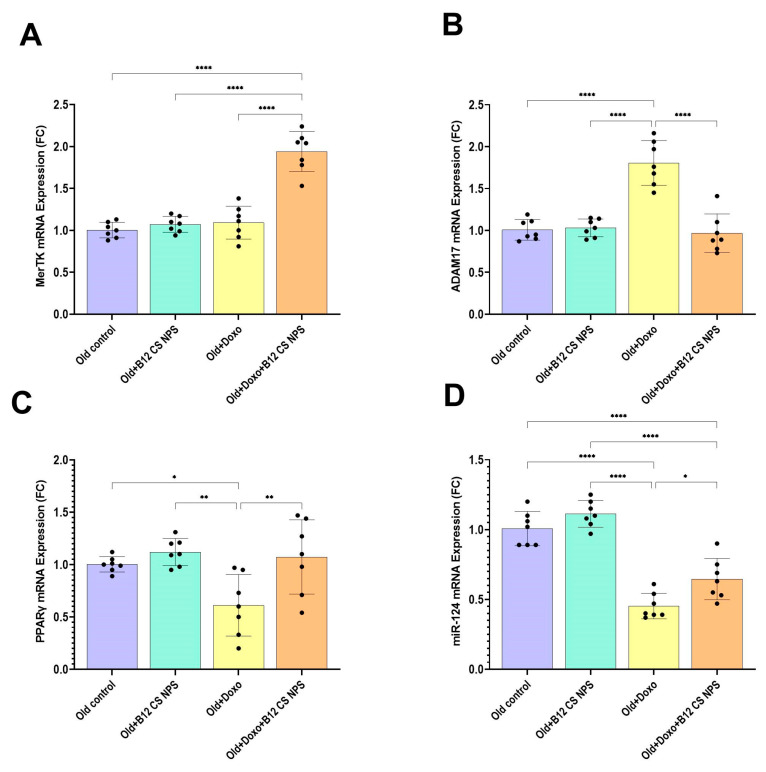
(**A**) MerTK, (**B**) ADAM17, (**C**) PPARγ, and (**D**) miR-124 gene expression in the gastrocnemius muscle tissue samples from experimental groups (*n* = 7/group), as determined by qPCR, normalized for the house-keeping gene B-actin, and expressed relative to controls. Results are expressed as mean ± SD. *, significant at (*p* < 0.05); **, significant at (*p* < 0.01), and ****, significant at (*p* < 0.0001).

We subsequently investigated the impact of Vit B12 on MerTK and ADAM17 mRNA expression. Upon Vit B12 administration, a notably enhanced MerTK (mean difference [old + Doxo–old + Doxo + B12 CS NPS] = −0.8486, 95% CI: −1.097 to −0.6005, *p* < 0.0001) (Figure 10A) and suppressed ADAM17 mRNA expression (mean difference [old + Doxo–old + Doxo + B12 CS NPS] = 0.8386, 95% CI: 0.5526 to 1.125, *p* < 0.0001) (Figure 10B) was observed in aged rats treated with Doxo + B12 CS NPS compared with aged rats treated with Doxo.

### 3.7. PPARγ and MiR-124 Are Enhanced by Vit B12 Administration

We next asked whether PPARγ and MiR-124 mRNA expressions are modulated by Vit B12 administration. Interestingly, Vit B12 supplementation successfully enhanced PPARγ (mean difference [old + Doxo–old + Doxo + B12 CS NPS] = −0.4614, 95% CI: −0.8184 to −0.1044, *p* < 0.01) (Figure 10C) and miR-124 expression (mean difference [old + Doxo–old + Doxo + B12 CS NPS] = −0.1929, 95% CI: −0.3647 to −0.02100, *p* < 0.05) (Figure 10D) in aged rats treated with Doxo+ B12 CS NPS compared with aged rats treated with Doxo. These data indicate that Vit B12 supplementation can be therapeutically effective at improving aging-impaired efferocytosis via modulating the molecular requirements governing MerTK and ADAM17 expression.

### 3.8. Vit B12-Enhanced Macrophage Phenotypic Shift Toward CD163^+^ M2 Phenotype at Day 3 Post-Doxorubicin-Induced Acute Myotoxicity

**Figure 11:**
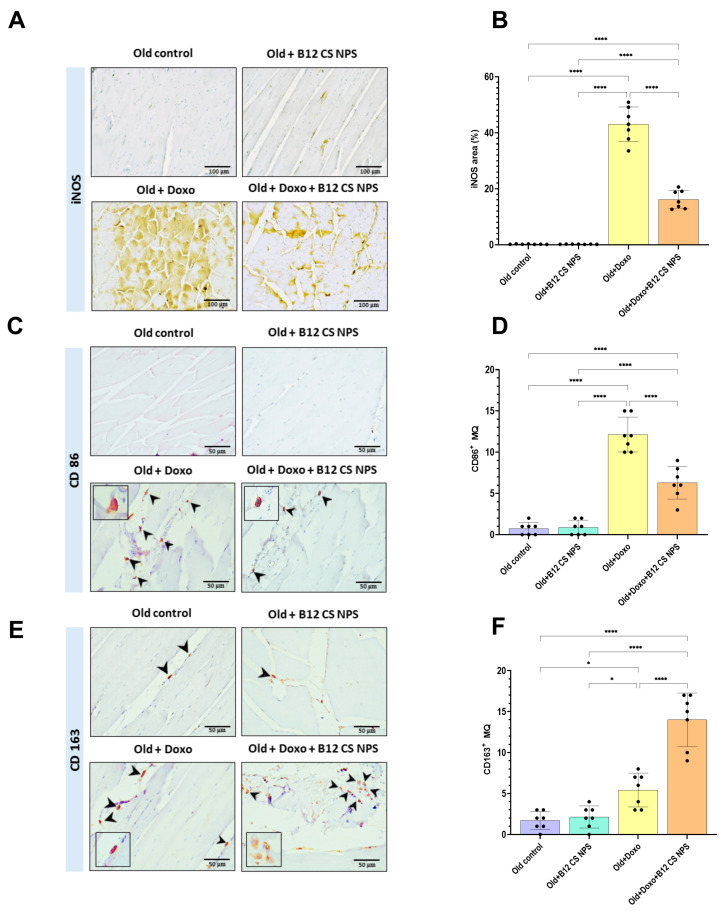
Immunohistochemical (IHC) detection of M1 and M2 polarization in skeletal muscles from different experimental groups, counterstained with hematoxylin. (**A**) Photoplate representing IHC detection of M1 macrophages based on anti-iNOS immune expression in all samples. (**B**) Quantitative representation of iNOS area%. (**C**) Photoplate representing IHC detection of M1 macrophages based on anti-CD86 immune expression in all samples. (**D**) Quantitative representation of CD86+ cells%. Little iNOS+ expression and few CD86+ M1 cells (arrowheads) are seen in Old + Doxo + B12-CS NPS group, indicating cessation of inflammation. ((**A**): scale bar = 100 μm, Magnification = ×200), ((**C**): scale bar = 50 μm, Magnification = ×400). (**E**) Detection of M2 macrophages based on anti-CD163 immune expression. The highest number of CD163^+^ M2 cells (arrowheads) are seen in Old + Doxo + B12 CS NPS group. Large number of large CD163^+^ M2 cells were distributed mainly in the endomysium and perimysium of muscle fibers and around the blood vessels. Note the large foamy appearance of M2 cells (small inset) representing phagocytic activity ((**E**): scale bar = 50 μm, Magnification = ×400). (**F**) Quantitative representation of CD163^+^ cells%. *, significant at (*p* < 0.05) and ****, significant at (*p* < 0.0001).

Macrophages are key for restoring muscle function post-injury and their phenotypic shift has been shown to regulate inflammation and tissue injury repair. We investigated whether macrophage phenotypic pattern is modulated by Vit B12 at this early time point. A significant increase in immunoreactivity of the M1 phenotype markers iNOS (mean difference [old + Doxo–old + Doxo + B12 CS NPS] = 26.88, 95% CI: 21.73 to 32.03, *p* < 0.0001) (Figure 11A,B) and CD86 (mean difference [old + Doxo–old + Doxo + B12 CS NPS] = 5.857, 95% CI: 3.554 to 8.161, *p* < 0.0001) (Figure 11C,D) was demonstrated in aged rats treated with Doxo compared with aged rats treated with Doxo + B12 CS NPS. Vit B12 administration was a potent inducer for polarization to the M2 phenotype, indicated by the significant increase in the immunoreactivity of the M2 phenotype marker CD163^+^ (mean difference [old + Doxo–old + Doxo + B12 CS NPS] = −8.571, 95% CI: −11.70 to −5.443, *p* < 0.0001) (Figure 11E,F) in aged rats treated with Doxo + B12 CS NPS compared with aged rats treated with Doxo.

### 3.9. Vit B12 Reduced Oxidative Stress and Inflammation, Promoted Extracellular Matrix Deposition, and Improved Remodeling Post-Doxorubicin-Induced Acute Myotoxicity

**Figure 12:**
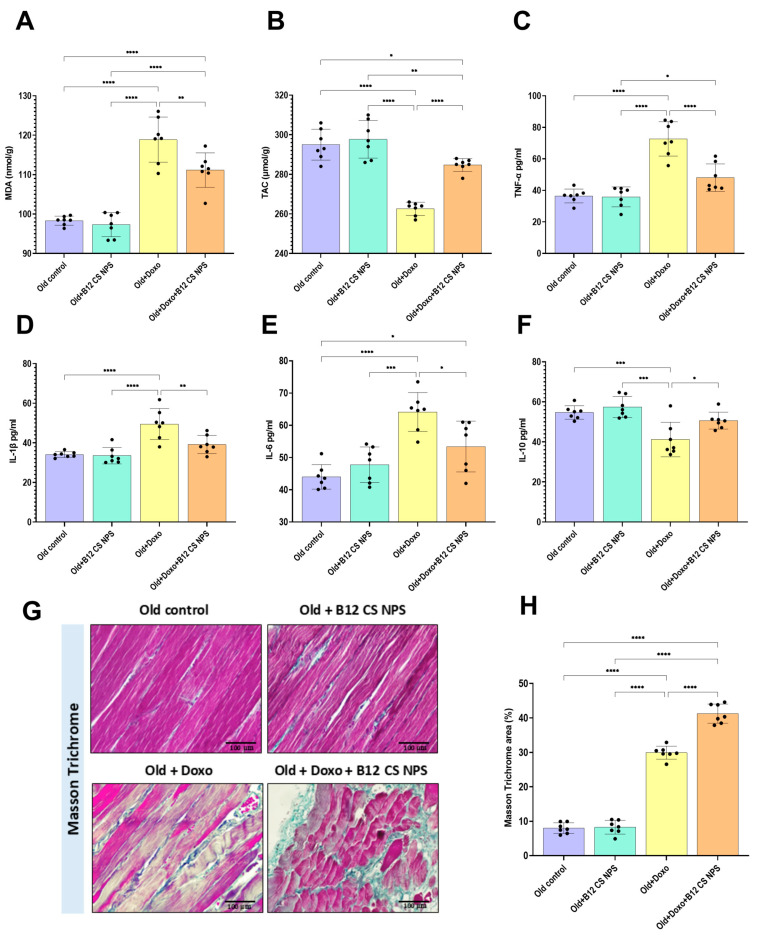
Effect of Vit B12 on oxidative stress, inflammatory markers and extracellular matrix deposition: (**A**) MDA, (**B**) TAC, (**C**) TNF-α, (**D**) IL-1β, (**E**) IL-6, (**F**) IL-10, (**G**) A photoplate representing collagen deposition in skeletal muscles. Detection of extracellular matrix collagen fibers stained with Masson’s trichrome stain. Green–blue color indicates positive results. (Scale bar = 100 μm, Magnification = ×200). (**H**) Analysis of positive Masson’s trichrome area%. *, significant at (*p* < 0.05); **, significant at (*p* < 0.01), ***, significant at (*p* < 0.001) and ****, significant at (*p* < 0.0001).

Aged rats treated with Doxo exhibited a significantly elevated levels of MDA (mean difference [old control–Old + Doxo] = −20.57, 95% CI: −26.40 to −14.74, *p* < 0.0001), as well as, a substantial decline in the TAC (mean difference [old control–Old + Doxo] = 32.43, 95% CI: 22.75 to 42.11, *p* < 0.0001) when compared with the control. Meanwhile, Vit B12 administration significantly reduced MDA levels (mean difference [old + Doxo—old + Doxo + B12 CS NPS] = 7.734, 95% CI: 1.903 to 13.57, *p* < 0.01) (Figure 12A) while raising the TAC (mean difference [old + Doxo–old + Doxo + B12 CS NPS] = −22.14, 95% CI: −31.82 to −12.46, *p* < 0.0001) (Figure 12B) in the aged rat group treated with Doxo + B12 CS NPS compared with aged rat group treated with Doxo.

Inflammatory cytokines released in response to muscle damage are precisely regulated. To assess the inflammatory response and whether Vit B12 modulates it at this time point, the pro-inflammatory TNFα, IL-1β, IL-6 and the anti-inflammatory IL-10 cytokines concentrations were measured. Aged rats treated with Doxo + B12 CS NPS exhibited a substantial decrease in TNFα (mean difference [old + Doxo–old + Doxo + B12 CS NPS] = 24.6, 95% CI: 12.84 to 36.36, *p* < 0.0001) (Figure 12C), IL-1β (mean difference [old + Doxo–old + Doxo + B12 CS NPS] = 10.34, 95% CI: 2.905 to 17.78, *p* < 0.01) (Figure 12D) and IL-6 (mean difference [old + Doxo–old + Doxo + B12 CS NPS] = 10.74, 95% CI: 1.916 to 19.57, *p* < 0.05) (Figure 12E) compared with aged rats treated with Doxo. In addition, a statistically significant elevation in IL-10 concentration was demonstrated in the aged rat group treated with Doxo + B12 CS NPS compared with the aged rat group treated with Doxo (mean difference [old + Doxo–old + Doxo + B12 CS NPS] = −9.414, 95% CI: −17.84 to −0.9875, *p* < 0.05) (Figure 12F). Together, these data demonstrated the antioxidant and the anti-inflammatory effect exerted by Vit B12 after acute muscle injury. Additionally, Vitamin B12 treatment significantly increased collagen fiber deposition following acute muscle injury. Both young and aged rats treated with doxorubicin showed elevated collagen levels compared to controls, with the highest expression observed in aged rat group treated with Doxo + B12 CS NPS (Figure 12G,H).

### 3.10. Vit B12 Enhanced Satellite Cell Activation and Early Differentiation at Day 3 Post-Doxorubicin-Induced Acute Myotoxicity

**Figure 13:**
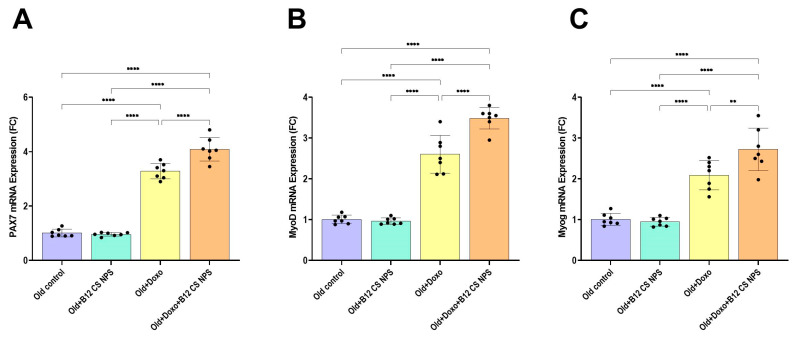
(**A**) Pax7, (**B**) MyoD, and (**C**) Myog gene expression in the gastrocnemius muscle tissue samples from experimental groups (*n* = 7/group), as determined by qPCR, normalized for the house-keeping gene B-actin, and expressed relative to controls. Results are expressed s at (*p* < 0.05); **, significant at (*p* < 0.001) and ****, significant at (*p* < 0.0001).

We focused our subsequent analysis on demonstrating the effect of Vit B12 on skeletal muscle regeneration. The mRNA levels of Pax7, the SC-specific transcription factor and the myogenic regulatory factors, MyoD and Myog were assessed in the gastrocnemius muscle tissues. Remarkably, supplementation with Vit B12 significantly enhanced the mRNA expression of Pax7 (mean difference [old + Doxo–old + Doxo + B12 CS NPS] = −0.8071, 95% CI: −1.208 to −0.4061, *p* < 0.0001) (Figure 13A), MyoD (mean difference [old + Doxo–old + Doxo + B12 CS NPS] = −0.8829, 95% CI: −1.288 to −0.4774, *p* < 0.0001) (Figure 13B), and Myog (mean difference [old + Doxo–old + Doxo + B12 CS NPS] = −0.6343, 95% CI: −1.119 to −0.1500, *p* < 0.01) (Figure 13C) on day 3 post-injury, indicating enhanced satellite cell proliferation and faster differentiation in the aged rat group treated with Doxo + B12 CS NPS compared with the aged rat group treated with Doxo. In accordance with the mRNA results, the histological examination of the aged rat group treated with Doxo + B12 CS NPS showed regenerative features including newly formed multiple myotubes formed outside the basal lamina with centrally aligned myocytes (↑), while some fibers showed internalized nuclei (arrowheads) and obvious inflammatory cells (dotted square) (Figure 4A(m–o)). Additionally, toluidine blue sections revealed active proliferating satellite cells differentiated to myocytes that aligned end-to-end forming new myotubes. These new fibers are characterized by the presence of internalized nuclei within the muscle fiber, a feature that distinguishes them from mature muscle fibers (Figure 6f–j). Furthermore, the ultrastructural examination supported these findings (Figure 9f,g).

## 4. Discussion

The ability of skeletal muscle to regenerate is governed by its crosstalk with immune cells. Immune cell functionality progressively declines with aging, contributing to impaired skeletal muscle regenerative ability. In addition, the emerging view of skeletal muscle as an immune-regulating tissue introduces additional complexity, as age-related muscle dysfunction may significantly disrupt the muscle–immune system crosstalk essential for effective tissue repair [34]. In particular, the impact of age on the phagocytosis-associated receptors and adaptors, molecules mediating apoptotic cell recognition in the muscle, and how this may influence skeletal tissue repair remains largely unclear. Therefore, in this study we first sought to unravel possible changes in the efferocytic machinery and its potential underlying regulatory molecules during aging.

Doxorubicin, a potent chemotherapeutic drug, is a well-established inducer of apoptosis in muscle cells [19,20,21], particularly in aging [22], due to enhanced mitochondrial production of reactive oxygen species (ROS). Therefore, we decided to study acute skeletal muscle injury repair during aging using this acute doxorubicin-induced skeletal muscle injury model. This model holds clinical relevance, as elucidating how aging impairs efferocytosis may uncover therapeutic strategies to enhance this process and mitigate doxorubicin-induced skeletal muscles toxicity. In this study, we demonstrate impaired efferocytosis in aged skeletal muscles after doxorubicin-induced acute myotoxicity. This finding aligns with emerging evidence linking aging to impaired efferocytosis. For example, De Maeyer et al. (2020) examined the impact of age on resolution and reported that the resolution phase, in a human dermal model of acute inflammation, was substantially impaired in elderly individuals due to reduced efferocytosis [35]. Hu et al. (2023) also found that aging impaired macrophages-mediated efferocytosis of apoptotic hepatocytes in mice subjected to liver ischemia–reperfusion (IR) injury [36].

Mechanistically, how aging impairs efferocytosis is not fully elucidated. Alterations in various elements of the efferocytosis machinery such as the TAM family of receptor tyrosine kinases and bridging molecules, through which efferocytes engage apoptotic cells (ACs), have been investigated in a number of studies with conflicting findings [6]. In particular, MerTK alterations and signaling have been studied in different settings. It has been reported that its cleavage in macrophages compromises repair after myocardial ischemia–reperfusion injury [37]. In addition, it was observed to undergo greater proteolytic cleavage in older mice in a remote lung injury model, resulting in impaired efferocytosis and delayed resolution of inflammation [38]. However, only a limited number of studies have examined the role of MerTK expression and its potential underlying regulatory molecules in efferocytosis in skeletal muscle injury during aging. We demonstrate that aged muscles experience suppressed MerTK associated with increased expression of ADAM17, a protease responsible for cleavage of the extracellular domain of the MerTK leading to defective efferocytosis [39,40]. In line with this result, Hu et al. (2023) demonstrated limited MerTK activity caused by enhanced ADAM17-mediated proteolytic cleavage in aged mice livers that experienced defected efferocytosis [36]. In contrast to rodents, there was no age-dependent difference in MerTK expression on human blood and blister monocytes/macrophages from a human dermal model of acute inflammation, while reduced TIM-4 expression was observed on mononuclear phagocytes (MPs) in elderly individuals and resulted in impaired clearance of apoptotic bodies [35]. These observations indicate that the aging process in rats and humans may involve different immunological changes and responses or that humans may rely on other pathways that preserve MerTK activity despite aging, which is a concept that needs further research.

To unravel new insights in the molecular mechanisms that could be impacted in the regulation of MerTK expression, we analyzed the expression of PPARγ and miR-124. PPARγ has previously been shown to be important for macrophage-mediated engulfment of apoptotic cells and the expression of a gene network implicated in phagocytosis [41,42]. PPARγ has also been reported to exert anti-inflammatory effects by binding miR-124 promoter and inducing its expression [12,43]. Moreover, miR-124 has emerged as a pivotal modulator of immune function and inflammation and has been shown to target ADAM17 mRNA, inhibiting its translation [44]. The present work revealed decreased PPARγ and miR-124 in aged rats treated with doxo. This result further confirms what has been previously reported by Rőszer et al. (2011) [41] and Luo et al. (2016) [42] regarding the role of PPARγ in enhancing MerTK expression and suggests that the associated decreased miR-124 contributes adversely to efferocytosis via enhancing ADAM17 mRNA translation. Enhanced ADAM17 increases MerTK surface shedding, leading to defective efferocytosis. Hence, this work uncovers an essential link between age-related impaired efferocytosis and the modulated expression of PPARγ and miR-124 during aging.

Recently, much attention has been paid to the role of Vit B12 in tissue repair [16,29], aging, and inflammation [45]; however, the underpinning molecular mechanisms are poorly known. In addition, studies investigating the immunomodulatory effect of Vit B12 are limited [17,46,47], with no previous studies having investigated its impact on efferocytosis. We therefore speculated that Vit B12 would help with tissue repair by improving age-related efferocytosis impairment.

Drug delivery to skeletal muscle tissue poses several challenges, including limited bioavailability, potential toxicity, and the large size of the target tissue. In this sense, nanoparticles-mediated drug delivery has been proposed as a promising alternative to allow therapeutic doses of drugs in the target tissue while reducing toxicity and enhancing pharmacokinetics and bioavailability. However, the application of nanostructures as therapeutic carriers in skeletal muscles remains limited, and research is largely confined to cellular models [48]. Additionally, skeletal muscle is often considered a tissue with limited targeting efficiency due to limited nanoparticle access under healthy physiological conditions; however, under pathological states and with aging, skeletal muscle vasculature—its structure, integrity, and permeability—is reported to be compromised [49,50], which encouraged us to consider Vit B12-loaded chitosan nanoparticles suitable for Vit B12 delivery into the skeletal muscle tissue in this context.

Our data demonstrate that Vit B12-loaded chitosan nanoparticle administration exerted an obvious enhancement of the efferocytic machinery and promoted the process of skeletal muscle injury repair. It seems that PPARγ is a key player in Vit B12-mediated restoration of defective efferocytosis, and subsequently enhanced tissue repair. We noted that Vit B12-loaded chitosan nanoparticle administration significantly increased PPARγ expression. Previous studies have highlighted the importance of PPARγ in Vit B12-mediated cell signaling and shown that impaired PPAR signaling is a hallmark of Vit B12 deficiency [51,52,53]. Consistent with the increased levels of PPARγ mediated by Vit B12 supplementation, the expression of MerTK was significantly increased. In support of this, Chen et al. (2015) showed that activating PPARγ accelerated timely disposal of apoptotic cells through increased expression of phagocytosis-related molecules, especially CD36 and Mertk [10]. More recently, Zheng et al. (2022) [54] and Zhuang et al. (2021) [55] also reported that PPAR-γ may act as an upstream regulator of Mertk. Along with the enhancement in PPARγ expression, miR-124 was significantly increased with Vit B12 supplementation. This implies that Vit B12 may rely more on both PPARγ and miR-124 to enhance mertk expression and negatively regulate ADAM17 with subsequent improvement in efferocytosis. Given that ROS has been proposed to activate ADAM17 [36,39,56], Vit B12’s power to increase the antioxidant capacity observed in this study, evidenced by reduced MDA levels and elevated TAC, is another significant finding that supports its inhibitory effect on ADAM17. Collectively, these findings outline a coordinated pathway through which Vitamin B12-loaded chitosan nanoparticles restore efferocytic signaling and promote skeletal muscle regeneration in aged rats following doxorubicin-induced injury. In line with our findings, Chen et al. (2024) demonstrated the ability of Vit B12 as a therapeutic agent to prevent the necrosis of acinar cells and restores the pancreatic structures in patients with acute pancreatitis [57].

Since there is mounting evidence that apoptotic cells clearance and M1-to-M2 transition are interconnected and constitute critical steps for muscle healing [7,58], we focused our subsequent analysis on the potential effect of Vit B12 supplementation on macrophage phenotypes polarization and the inflammatory status at this time point. Remarkably, supplementation with Vit B12 was a potent inducer for polarization to the M2 phenotype at day 3 post-injury, indicating a delay in the appearance of M2 macrophage in the aged rat group treated with doxo. The enhanced appearance of M2-biased macrophage with Vit B12 administration was coupled with a significant increase in the expression of the anti-inflammatory cytokine IL-10 and a substantial decrease in the inflammatory cytokines TNFα, IL-1β and IL-6. It is important to note that at this time point, markers of both M1 and M2 phenotypes were expressed, but the predominance of M2-biased phenotype was observed in rats administered Vit B12. Consistent with our results, previous studies have shown that efferocytosis fosters a pro-resolving phenotype by suppressing pro-inflammatory cytokine, upregulating pro-resolving mediators, downregulating inducible nitric oxide synthase (iNOS), and enhancing angiogenic growth factors production [7,59,60,61]. Additionally, it has been reported that during efferoctosis, MerTK blocks NF- κB signaling in macrophages to suppress inflammatory cytokine production and macrophage M1-like polarization [62,63] and enhance M2-like phenotype [64,65], which further supports our findings.

It has been established that the dynamic shift in macrophages across phenotypes during muscle regeneration is crucial for successful repair [3,58,66]. Satellite cell differentiation and maturation of new myofibres are facilitated by the transition to an anti-inflammatory microenvironment promoted by M2 macrophages [4,67]. In accordance with that, the observed predominance of M2-biased macrophage with Vit B12 supplementation was concomitant with a significant increase in the mRNA expression of Pax7, MyoD, and Myog, indicating enhanced satellite cell proliferation and faster differentiation. In line with our findings, Li et al. (2022) reported that Cyanocobalamin (Vitamin B12) enhances the proliferation and maturation of muscle satellite cells, thereby facilitating muscle regeneration [29].

On the contrary, Nawaz et al. (2022) reported that myogenesis is promoted by the depletion of CD206+ M2-like macrophages [68]. These apparently conflicting results are likely attributed to different models of skeletal muscle injury, or different time points after injury at which tissue samples were examined. Thus, more research is required for the evaluation of CD206+ M2-like macrophages and their role during muscle regeneration.

Several limitations and future directions should be acknowledged. While our study shows that Vit B12 enhances efferocytosis and M2 macrophage polarization, the role of the MerTK/PPARγ/miR-124 axis remains correlative, and other pathways may contribute to these effects. Additionally, direct phagocytic quantification and mechanistic validation using pathway-specific inhibition or rescue experiments were not performed. Long-term regenerative outcomes and the durability of Vit B12 effects were not assessed. The pharmacokinetics and tissue availability of free vitamin B12 were not directly measured, and optimal dosing and delivery require further study. With respect to Vit B12 nano formulation, this study focused on initial physicochemical characterization to support the in vivo efficacy, and thus did not include long-term stability profiling or extended-release kinetics of the Vit B12-loaded chitosan nanoparticles. In addition, systemic safety, biodistribution, and potential off-target effects of the nano formulation were not assessed in this study. Although both Vit B12 and chitosan are widely regarded as safe and biocompatible, nano formulation can alter pharmacokinetics, cellular uptake, and tissue retention compared to free compounds. Therefore, future studies will evaluate stability under different storage conditions and assess sustained release behavior to better determine translational feasibility and include comprehensive biodistribution analyses and long-term safety evaluation to confirm tolerability and support translational development.

## 5. Conclusions

In summary, our results support the notion that aging impairs efferocytosis and advances our current molecular understanding of how aging may impair the efferocytic process in skeletal muscles after injury. We demonstrate MerTK, ADAM17, PPARγ, and miR-124 as regulatory molecules of the efferocytosis process that are altered with aging. Moreover, we highlight the emerging link between boosting efferocytosis, skewing macrophage polarization to the anti-inflammatory phenotype, promoting resolution and the improvement in the regenerative capacity of skeletal muscle after injury and emphasize Vit B12-loaded chitosan nanoparticles’ role in enhancing the efferocytic machinery and promoting the process of skeletal muscle injury repair.

## Figures and Tables

**Table 1:** Primer sequences utilized for qPCR analysis.

Gene Name	Primer Sequences (5′→3′) (F: Forward; R: Reverse)	Accession Number
*Bax*	F: TGGCCTCCTTTCCTACTTCG R: AAAATGCCTTTCCCCGTTCC	NM_017059.2
*Bcl-2*	F: AACTCTTCAGGGATGGGGTG R: GCTGGGGCCATATAGTTCCA	NM_016993.2
*MerTK*	F: AGGGTTTGATGGCTACTCCC R: ACCAGCCAATCTCATTCCGA	NM_022943.1
*ADAM17*	F: CTGTGCCTTGTCTCTCCTGA R: TACATACACCCACACACCCC	NM_020306.3
*miR-124*	F: TCAAGATCAGAGACTCTGCTC R: TTCAAGTGCAGCCGTAGG	NR_031867.1
*PPARγ*	F: GGATTCATGACCAGGGAGTTCCTC R: GCGGTCTCCACTGAGAATAATGAC	NM_013124.3
*Myog*	F: GAGCCCCACTTCTATGACGG R: GTTGAGCAGGGTGCTTCTCT	NM_017115.3
*PAX7*	F: AGCCGAGTGCTCAGAATCAA R: TCCTCTCGAAAGCCTTCTCC	NM_001191984.2
*MyoD*	F: GACGGCTCTCTCTGCTCCTT R: GTCTGAGTCGCCGCTGTAGT	NM_176079.2
*B-actin*	F: AACCTTCTTGCAGCTCCTCC R: CCATACCCACCATCACACCC	NM_031144.3

## Data Availability

The original contributions presented in this study are included in the article/Supplementary Material. Further inquiries can be directed to the corresponding author.

## Supplementary Materials

The following supporting information can be downloaded at: https://www.mdpi.com/article/10.3390/biom15121709/s1, Figure S1: Correlations between MerTK expression and inflammatory/oxidative stress markers in old groups. Scatter plots showing the relationship between MerTK expression and selected inflammatory and oxidative stress markers in skeletal muscle following doxorubicin-induced acute myotoxicity. Significant positive correlations were observed between MerTK and CD163 and between MerTK and iNOS. No significant correlations were detected with other measured markers. Correlation coefficients (r) and p-values are indicated for each plot; Figure S2. Correlations between ADAM17 expression and inflammatory/oxidative stress markers in old groups. Scatter plots showing the relationship between ADAM17 expression and selected inflammatory and oxidative stress markers in skeletal muscle following doxorubicin-induced acute myotoxicity. ADAM17 expression was positively correlated with TNF-α, iNOS, IL-6, CD86, and MDA, and negatively correlated with TAC and IL-10. Correlation coefficients (r) and p-values are indicated for each plot.

## Abbreviations

The following abbreviations are used in this manuscript: B12 CS NPS: Vitamin B12-loaded chitosan nanoparticles; DOXO: Doxorubicin; Myog: Myogenin; MerTK: Mer tyrosine kinase; ADAM17: A disintegrin and metalloproteinase 17; TACE: TNF-α converting enzyme; TNF-α: Tumor necrosis factor alpha; PPARγ: Peroxisome proliferator-activated receptor γ; IL-1β: Interleukin-1β; MDA: Malondialdehyde; TAC: Total antioxidant capacity; FAPs: Fibro–adipogenic progenitor cells.

## References

[1] Ocampo A., Reddy P., Martinez-Redondo P., Platero-Luengo A., Hatanaka F., Hishida T., Li M., Lam D., Kurita M., Beyret E. (2016). In Vivo Amelioration of Age-Associated Hallmarks by Partial Reprogramming. Cell.

[2] Muñoz-Cánoves P., Neves J., Sousa-Victor P. (2020). Understanding muscle regenerative decline with aging: New approaches to bring back youthfulness to aged stem cells. FEBS J..

[3] Tidball J.G., Flores I., Welc S.S., Wehling-Henricks M., Ochi E. (2021). Aging of the immune system and impaired muscle regeneration: A failure of immunomodulation of adult myogenesis. Exp. Gerontol..

[4] Perandini L.A., Chimin P., Lutkemeyer D.D.S., Câmara N.O.S. (2018). Chronic inflammation in skeletal muscle impairs satellite cells function during regeneration: Can physical exercise restore the satellite cell niche?. FEBS J..

[5] Juban G., Chazaud B. (2021). Efferocytosis during Skeletal Muscle Regeneration. Cells.

[6] Poon I.K.H., Ravichandran K.S. (2024). Targeting Efferocytosis in Inflammaging. Annu. Rev. Pharmacol. Toxicol..

[7] Doran A.C., Yurdagul A., Tabas I. (2020). Efferocytosis in health and disease. Nat. Rev. Immunol..

[8] Al-Zaeed N., Budai Z., Szondy Z., Sarang Z. (2021). TAM kinase signaling is indispensable for proper skeletal muscle regeneration in mice. Cell Death Dis..

[9] Lahey K.C., Varsanyi C., Wang Z., Aquib A., Gadiyar V., Rodrigues A.A., Pulica R., Desind S., Davra V., Calianese D.C. (2024). Regulation of Mertk Surface Expression via ADAM17 and γ-Secretase Proteolytic Processing. Int. J. Mol. Sci..

[10] Chen H., Shi R., Luo B., Yang X., Qiu L., Xiong J., Jiang M., Liu Y., Zhang Z., Wu Y. (2015). Macrophage peroxisome proliferator-activated receptor γ deficiency delays skin wound healing through impairing apoptotic cell clearance in mice. Cell Death Dis..

[11] Hou Z., Chen J., Yang H., Hu X., Yang F. (2021). PIAS1 alleviates diabetic peripheral neuropathy through SUMOlation of PPAR-γ and miR-124-induced downregulation of EZH2/STAT3. Cell Death Discov..

[12] Wang D., Shi L., Xin W., Xu J., Xu J., Li Q., Xu Z., Wang J., Wang G., Yao W. (2017). Activation of PPARγ inhibits pro-inflammatory cytokines production by upregulation of miR-124 in vitro and in vivo. Biochem. Biophys. Res. Commun..

[13] Calligaris M., Cuffaro D., Bonelli S., Spanò D.P., Rossello A., Nuti E., Scilabra S.D. (2021). Strategies to Target ADAM17 in Disease: From Its Discovery to the iRhom Revolution. Molecules.

[14] Qin Z., Wang P.-Y., Su D.-F., Liu X. (2016). miRNA-124 in Immune System and Immune Disorders. Front. Immunol..

[15] Artimovič P., Špaková I., Macejková E., Pribulová T., Rabajdová M., Mareková M., Zavacká M. (2024). The ability of microRNAs to regulate the immune response in ischemia/reperfusion inflammatory pathways. Genes Immun..

[16] Kovatcheva M., Melendez E., Chondronasiou D., Pietrocola F., Bernad R., Caballe A., Junza A., Capellades J., Holguín-Horcajo A., Prats N. (2023). Vitamin B12 is a limiting factor for induced cellular plasticity and tissue repair. Nat. Metab..

[17] Tamura J., Kubota K., Murakami H., Sawamura M., Matsushima T., Tamura T., Saitoh T., Kurabayshi H., Naruse T. (2001). Immunomodulation by vitamin B12: Augmentation of CD8^+^ T lymphocytes and natural killer (NK) cell activity in vitamin B12-deficient patients by methyl-B12 treatment. Clin. Exp. Immunol..

[18] Ge Y., Yang C., Zadeh M., Sprague S.M., Lin Y.D., Jain H.S., Determann B.F., Roth W.H., Palavicini J.P., Larochelle J. (2024). Functional regulation of microglia by vitamin B12 alleviates ischemic stroke-induced neuroinflammation in mice. iScience.

[19] Min K., Kwon O.S., Smuder A.J., Wiggs M.P., Sollanek K.J., Christou D.D., Yoo J.K., Hwang M.H., Szeto H.H., Kavazis A.N. (2015). Increased mitochondrial emission of reactive oxygen species and calpain activation are required for doxorubicin-induced cardiac and skeletal muscle myopathy. J. Physiol..

[20] Yu A.P., Pei X.M., Sin T.K., Yip S.P., Yung B.Y., Chan L.W., Wong C.S., Siu P.M. (2014). Acylated and unacylated ghrelin inhibit doxorubicin-induced apoptosis in skeletal muscle. Acta Physiol..

[21] Hiensch A.E., Bolam K.A., Mijwel S., Jeneson J.A.L., Huitema A.D.R., Kranenburg O., van der Wall E., Rundqvist H., Wengstrom Y., May A.M. (2020). Doxorubicin-induced skeletal muscle atrophy: Elucidating the underlying molecular pathways. Acta Physiol..

[22] Sin T.K., Tam B.T., Yu A.P., Yip S.P., Yung B.Y., Chan L.W., Wong C.S., Rudd J.A., Siu P.M. (2016). Acute Treatment of Resveratrol Alleviates Doxorubicin-Induced Myotoxicity in Aged Skeletal Muscle Through SIRT1-Dependent Mechanisms. J. Gerontol. Ser. A Biol. Sci. Med. Sci..

[23] Guler E., Yekeler H.B., Parviz G., Aydin S., Asghar A., Dogan M., Ikram F., Kalaskar D.M., Cam M.E. (2024). Vitamin B12-loaded chitosan-based nanoparticle-embedded polymeric nanofibers for sublingual and transdermal applications: Two alternative application routes for vitamin B12. Int. J. Biol. Macromol..

[24] Abd El-Emam M.M., Behairy A., Mostafa M., Khamis T., Osman N.M.S., Alsemeh A.E., Mansour M.F. (2024). Chrysin-loaded PEGylated liposomes protect against alloxan-induced diabetic neuropathy in rats: The interplay between endoplasmic reticulum stress and autophagy. Biol. Res..

[25] Reeves P.G., Nielsen F.H., Fahey G.C. (1993). AIN-93 purified diets for laboratory rodents: Final report of the American Institute of Nutrition ad hoc writing committee on the reformulation of the AIN-76A rodent diet. J. Nutr..

[26] National Research Council (2011). Guide for the Care and Use of Laboratory Animals.

[27] Smuder A.J., Kavazis A.N., Min K., Powers S.K. (2011). Exercise protects against doxorubicin-induced oxidative stress and proteolysis in skeletal muscle. J. Appl. Physiol..

[28] Huo M., Tang Z., Wang L., Zhang L., Guo H., Chen Y., Gu P., Shi J. (2022). Magnesium hexacyanoferrate nanocatalysts attenuate chemodrug-induced cardiotoxicity through an anti-apoptosis mechanism driven by modulation of ferrous iron. Nat. Commun..

[29] Li P., Zhao Y., Liu Y., Zhao Y., Yan Y., Li S., Li S., Tong H. (2022). Cyanocobalamin promotes muscle development through the TGF-β signaling pathway. Food Funct..

[30] Livak K.J., Schmittgen T.D. (2001). Analysis of Relative Gene Expression Data Using Real-Time Quantitative PCR and the 2−ΔΔCT Method. Methods.

[31] Bancroft J.D., Layton C. (2019). The hematoxylin and eosin stain. Bancroft’s Theory and Practice of Histological Techniques.

[32] Mondal S.K. (2017). Manual of Histological Techniques.

[33] Glauert A.M., Lewis P.R. (1998). Embedding methods. Biological Specimen Preparation for Transmission Electron Microscopy.

[34] Nelke C., Dziewas R., Minnerup J., Meuth S.G., Ruck T. (2019). Skeletal muscle as potential central link between sarcopenia and immune senescence. eBioMedicine.

[35] De Maeyer R.P.H., van de Merwe R.C., Louie R., Bracken O.V., Devine O.P., Goldstein D.R., Uddin M., Akbar A.N., Gilroy D.W. (2020). Blocking elevated p38 MAPK restores efferocytosis and inflammatory resolution in the elderly. Nat. Immunol..

[36] Hu H., Cheng X., Li F., Guan Z., Xu J., Wu D., Gao Y., Zhan X., Wang P., Zhou H. (2023). Defective efferocytosis by aged macrophages promotes STING signaling mediated inflammatory liver injury. Cell Death Discov..

[37] DeBerge M., Yeap X.Y., Dehn S., Zhang S., Grigoryeva L., Misener S., Procissi D., Zhou X., Lee D.C., Muller W.A. (2017). MerTK Cleavage on Resident Cardiac Macrophages Compromises Repair After Myocardial Ischemia Reperfusion Injury. Circ. Res..

[38] Rymut N., Heinz J., Sadhu S., Hosseini Z., Riley C.O., Marinello M., Maloney J., MacNamara K.C., Spite M., Fredman G. (2020). Resolvin D1 promotes efferocytosis in aging by limiting senescent cell-induced MerTK cleavage. FASEB J..

[39] Thorp E., Vaisar T., Subramanian M., Mautner L., Blobel C., Tabas I. (2011). Shedding of the Mer Tyrosine Kinase Receptor Is Mediated by ADAM17 Protein through a Pathway Involving Reactive Oxygen Species, Protein Kinase Cδ, and p38 Mitogen-activated Protein Kinase (MAPK). J. Biol. Chem..

[40] Sather S., Kenyon K.D., Lefkowitz J.B., Liang X., Varnum B.C., Henson P.M., Graham D.K. (2007). A soluble form of the Mer receptor tyrosine kinase inhibits macrophage clearance of apoptotic cells and platelet aggregation. Blood.

[41] Rőszer T., Menéndez-Gutiérrez M.P., Lefterova M.I., Alameda D., Núñez V., Lazar M.A., Fischer T., Ricote M. (2011). Autoimmune Kidney Disease and Impaired Engulfment of Apoptotic Cells in Mice with Macrophage Peroxisome Proliferator-Activated Receptor γ or Retinoid X Receptor α Deficiency. J. Immunol..

[42] Luo B., Gan W., Liu Z., Shen Z., Wang J., Shi R., Liu Y., Liu Y., Jiang M., Zhang Z. (2016). Erythropoeitin Signaling in Macrophages Promotes Dying Cell Clearance and Immune Tolerance. Immunity.

[43] El Gazzar W., Allam M., Shaltout S., Mohammed L., Sadek A., Nasr H. (2022). Pioglitazone modulates immune activation and ameliorates inflammation induced by injured renal tubular epithelial cells via PPARγ/miRNA-124/STAT3 signaling. Biomed. Rep..

[44] Sun Y., Li Q., Gui H., Xu D.-P., Yang Y.-L., Su D.-F., Liu X. (2013). MicroRNA-124 mediates the cholinergic anti-inflammatory action through inhibiting the production of pro-inflammatory cytokines. Cell Res..

[45] Simonenko S.Y., Bogdanova D.A., Kuldyushev N.A. (2024). Emerging Roles of Vitamin B12 in Aging and Inflammation. Int. J. Mol. Sci..

[46] Erkurt M.A., Aydogdu I., Dikilitaş M., Kuku I., Kaya E., Bayraktar N., Ozhan O., Ozkan I., Sönmez A. (2008). Effects of Cyanocobalamin on Immunity in Patients with Pernicious Anemia. Med. Princ. Pract..

[47] Ge Y., Zadeh M., Sharma C., Lin Y.-D., Soshnev A.A., Mohamadzadeh M. (2024). Controlling functional homeostasis of ileal resident macrophages by vitamin B12 during steady state and Salmonella infection in mice. Mucosal Immunol..

[48] Colapicchioni V., Millozzi F., Parolini O., Palacios D. (2022). Nanomedicine, a valuable tool for skeletal muscle disorders: Challenges, promises, and limitations. WIREs Nanomed. Nanobiotechnol..

[49] Hicks M.R., Liu X., Young C.S., Saleh K., Ji Y., Jiang J., Emami M.R., Mokhonova E., Spencer M.J., Meng H. (2023). Nanoparticles systemically biodistribute to regenerating skeletal muscle in DMD. J. Nanobiotechnol..

[50] Oakley R. (2014). Vascular Hyperpermeability and Aging. Aging Dis..

[51] Ge Y., Zadeh M., Mohamadzadeh M. (2022). Vitamin B12 Regulates the Transcriptional, Metabolic, and Epigenetic Programing in Human Ileal Epithelial Cells. Nutrients.

[52] Ahmad S., Kumar K.A., Basak T., Bhardwaj G., Yadav D.K., Lalitha A., Chandak G.R., Raghunath M., Sengupta S. (2013). PPAR signaling pathway is a key modulator of liver proteome in pups born to vitamin B12 deficient rats. J. Proteom..

[53] Meher A., Joshi A., Joshi S. (2014). Differential Regulation of Hepatic Transcription Factors in the Wistar Rat Offspring Born to Dams Fed Folic Acid, Vitamin B12 Deficient Diets and Supplemented with Omega-3 Fatty Acids. PLoS ONE.

[54] Zheng L., Jia J., Chen Y., Liu R., Cao R., Duan M., Zhang M., Xu Y. (2022). Pentoxifylline alleviates ischemic white matter injury through up-regulating Mertk-mediated myelin clearance. J. Neuroinflamm..

[55] Zhuang J., Peng Y., Gu C., Chen H., Lin Z., Zhou H., Wu X., Li J., Yu X., Cao Y. (2021). Wogonin Accelerates Hematoma Clearance and Improves Neurological Outcome via the PPAR-γ Pathway After Intracerebral Hemorrhage. Transl. Stroke Res..

[56] Zhang Y., Wang Y., Zhou D., Zhang L.S., Deng F.X., Shu S., Wang L.J., Wu Y., Guo N., Zhou J. (2019). Angiotensin II deteriorates advanced atherosclerosis by promoting MerTK cleavage and impairing efferocytosis through the AT 1 R/ROS/p38 MAPK/ADAM17 pathway. Am. J. Physiol.-Cell Physiol..

[57] Chen Y., Li X., Lu R., Lv Y., Wu Y., Ye J., Zhao J., Li L., Huang Q., Meng W. (2024). Vitamin B 12 protects necrosis of acinar cells in pancreatic tissues with acute pancreatitis. MedComm.

[58] Sciorati C., Rigamonti E., Manfredi A.A., Rovere-Querini P. (2016). Cell death, clearance and immunity in the skeletal muscle. Cell Death Differ..

[59] Noelia A., Bensinger S.J., Hong C., Beceiro S., Bradley M.N., Zelcer N., Deniz J., Ramirez C., Díaz M., Gallardo G. (2009). Apoptotic Cells Promote Their Own Clearance and Immune Tolerance through Activation of the Nuclear Receptor LXR. Immunity.

[60] Ariel A., Serhan C.N. (2012). New Lives Given by Cell Death: Macrophage Differentiation Following Their Encounter with Apoptotic Leukocytes during the Resolution of Inflammation. Front. Immunol..

[61] Mukundan L., Odegaard J.I., Morel C.R., Heredia J.E., Mwangi J.W., Ricardo-Gonzalez R.R., Goh Y.S., Eagle A.R., Dunn S.E., Awakuni J.U. (2009). PPAR-δ senses and orchestrates clearance of apoptotic cells to promote tolerance. Nat. Med..

[62] Mehrotra P., Ravichandran K.S. (2022). Drugging the efferocytosis process: Concepts and opportunities. Nat. Rev. Drug Discov..

[63] Tibrewal N., Wu Y., D’mello V., Akakura R., George T.C., Varnum B., Birge R.B. (2008). Autophosphorylation Docking Site Tyr-867 in Mer Receptor Tyrosine Kinase Allows for Dissociation of Multiple Signaling Pathways for Phagocytosis of Apoptotic Cells and Down-modulation of Lipopolysaccharide-inducible NF-κB Transcriptional Activation. J. Biol. Chem..

[64] Filardy A.A., Pires D.R., Nunes M.P., Takiya C.M., Freire-de-Lima C.G., Ribeiro-Gomes F.L., DosReis G.A. (2010). Proinflammatory Clearance of Apoptotic Neutrophils Induces an IL-12lowIL-10high Regulatory Phenotype in Macrophages. J. Immunol..

[65] Zizzo G., Hilliard B.A., Monestier M., Cohen P.L. (2012). Efficient Clearance of Early Apoptotic Cells by Human Macrophages Requires M2c Polarization and MerTK Induction. J. Immunol..

[66] Wang X., Zhou L. (2022). The Many Roles of Macrophages in Skeletal Muscle Injury and Repair. Front. Cell Dev. Biol..

[67] Careccia G., Mangiavini L., Cirillo F. (2023). Regulation of Satellite Cells Functions during Skeletal Muscle Regeneration: A Critical Step in Physiological and Pathological Conditions. Int. J. Mol. Sci..

[68] Nawaz A., Bilal M., Fujisaka S., Kado T., Aslam M.R., Ahmed S., Okabe K., Igarashi Y., Watanabe Y., Kuwano T. (2022). Depletion of CD206^+^ M2-like macrophages induces fibro-adipogenic progenitors activation and muscle regeneration. Nat. Commun..

