# Supplementary material for: Vitamin B12-Loaded Chitosan Nanoparticles Promote Skeletal Muscle Injury Repair in Aged Rats via Amelioration of Aging-Suppressed Efferocytosis
Source: Biomolecules. 2025 Dec 7;15(12):1709. doi: 10.3390/biom15121709 (PMC12730411; doi:10.3390/biom15121709)
Supplement: Supplementary file 1 [file biomolecules-15-01709-s001.zip › biomolecules-3984086-supplementary.pdf]

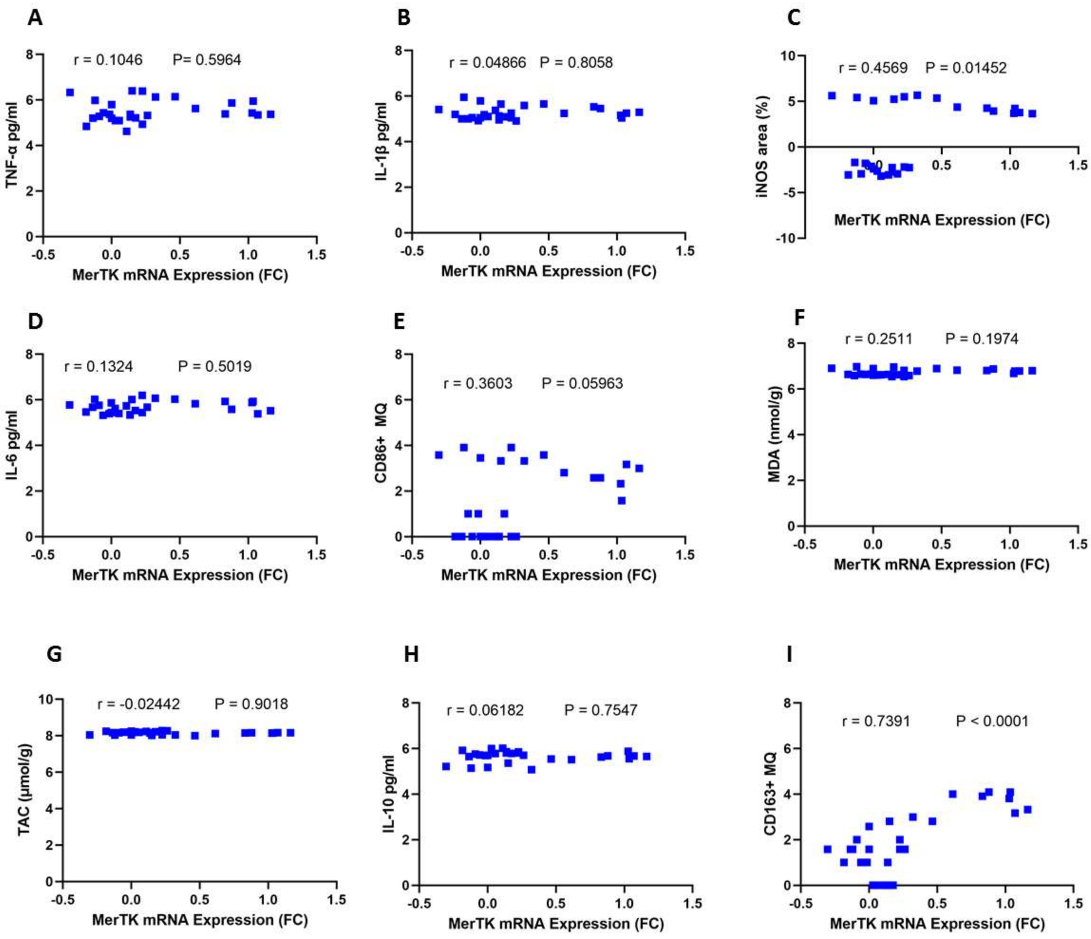

Figure S1. Correlations between MerTK expression and inflammatory/oxidative stress markers in old groups. Scatter plots showing the relationship between MerTK expression and selected inflammatory and oxidative stress markers in skeletal muscle following doxorubicin-induced acute myotoxicity. Significant positive correlations were observed between MerTK and CD163 and between MerTK and iNOS. No significant correlations were detected with other measured markers. Correlation coefficients (r) and p-values are indicated for each plot.

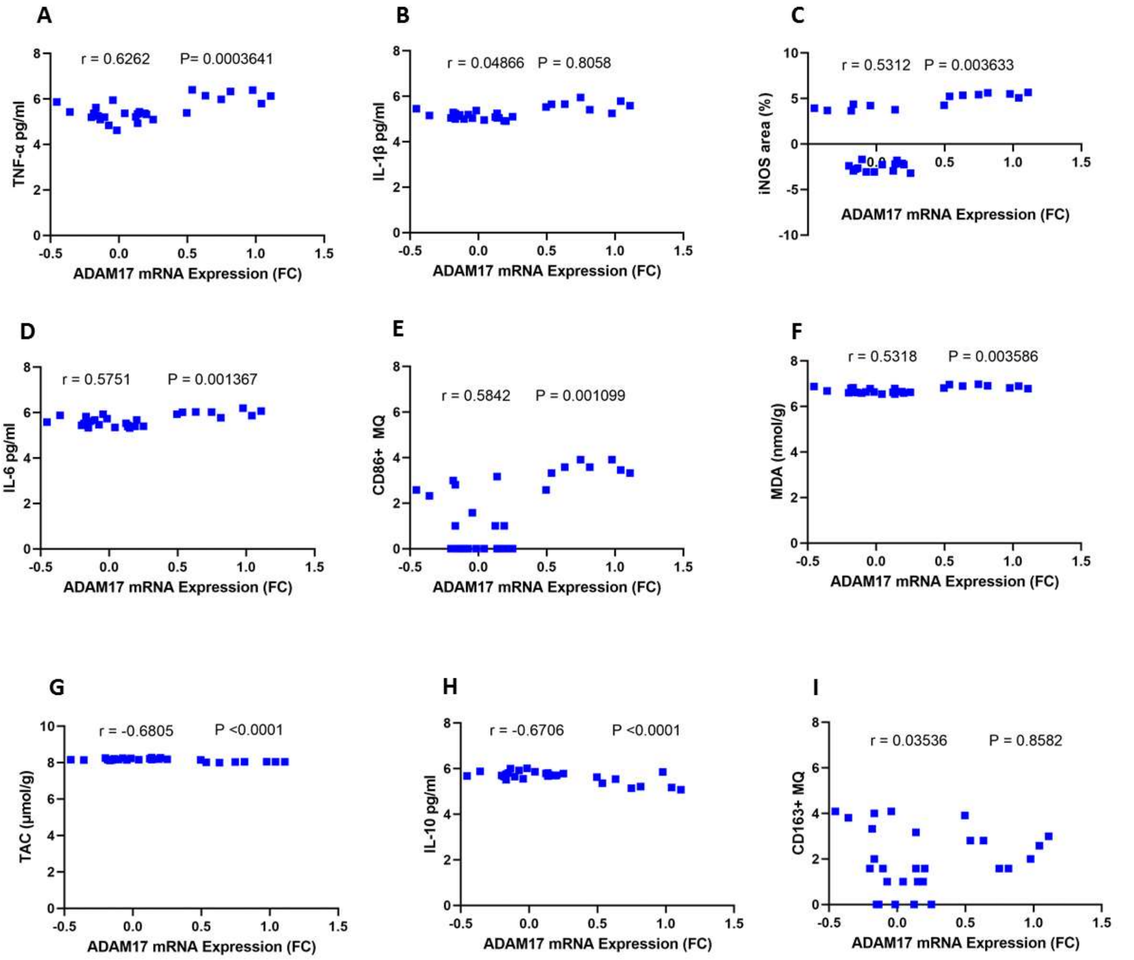

Figure S2. Correlations between ADAM17 expression and inflammatory/oxidative stress markers in old groups. Scatter plots showing the relationship between ADAM17 expression and selected inflammatory and oxidative stress markers in skeletal muscle following doxorubicin-induced acute myotoxicity. ADAM17 expression was positively correlated with TNF-α, iNOS, IL-6, CD86, and MDA, and negatively correlated with TAC and IL-10. Correlation coefficients (r) and p-values are indicated for each plot.
